# Graphene Nanostructure-Based Tactile Sensors for Electronic Skin Applications

**DOI:** 10.1007/s40820-019-0302-0

**Published:** 2019-09-04

**Authors:** Pei Miao, Jian Wang, Congcong Zhang, Mingyuan Sun, Shanshan Cheng, Hong Liu

**Affiliations:** 1 Institute for Advanced Interdisciplinary Research, Collaborative Innovation Center of Technology and Equipment for Biological Diagnosis and Therapy in Universities of Shandong, University of Jinan, 336 Nanxinzhuang West Road, Jinan, 250011 People’s Republic of China; 2 Department of Chemistry, School of Chemistry and Chemical Engineering, University of Jinan, 336 Nanxinzhuang West Road, Jinan, 250011 People’s Republic of China; 3 Department of Chemistry, Tianjin Key Laboratory of Molecular Optoelectronic Sciences, School of Science, Tianjin University, 92 Weijin Road, Tianjin, 300072 People’s Republic of China; 4 Center of Bio and Micro/Nano Functional Materials, State Key Laboratory of Crystal Materials, Shandong University, 27 Shanda South Road, Jinan, 250100 People’s Republic of China

**Keywords:** Graphene derivatives, Tactile sensor, Electronic skin, Assembly

## Abstract

Skin is the largest organ of the human body and can perceive and respond to complex environmental stimulations. Recently, the development of electronic skin (E-skin) for the mimicry of the human sensory system has drawn great attention due to its potential applications in wearable human health monitoring and care systems, advanced robotics, artificial intelligence, and human–machine interfaces. Tactile sense is one of the most important senses of human skin that has attracted special attention. The ability to obtain unique functions using diverse assembly processible methods has rapidly advanced the use of graphene, the most celebrated two-dimensional material, in electronic tactile sensing devices. With a special emphasis on the works achieved since 2016, this review begins with the assembly and modification of graphene materials and then critically and comprehensively summarizes the most advanced material assembly methods, device construction technologies and signal characterization approaches in pressure and strain detection based on graphene and its derivative materials. This review emphasizes on: (1) the underlying working principles of these types of sensors and the unique roles and advantages of graphene materials; (2) state-of-the-art protocols recently developed for high-performance tactile sensing, including representative examples; and (3) perspectives and current challenges for graphene-based tactile sensors in E-skin applications. A summary of these cutting-edge developments intends to provide readers with a deep understanding of the future design of high-quality tactile sensing devices and paves a path for their future commercial applications in the field of E-skin.

## Highlights

- Tremendous progress has been advanced by research into graphene and its derivatives with great benefits toward low-cost, portable, and real-time tactile sensors/electronic skin.
- The review presented herein direct future efforts aimed at high-quality graphene-based tactile sensors and their implications for the wider scientific community.
- The paper also are informative regarding some basic and crucial issues regarding graphene and its derivatives, such as charge-transport principles, doping/trapping behaviors, correlations between structure/morphology and properties/functions.

## Graphical abstract

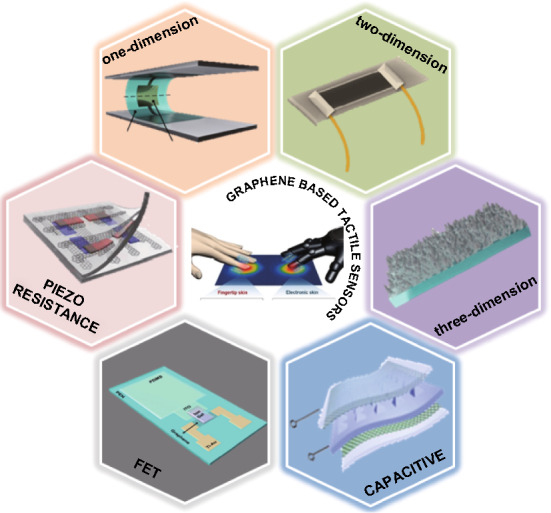

## Introduction

A tactile sensor is a kind of device that simulates the tactile sense of human skin and can detect and analyze the strength, position and time sequence of an external mechanical force with micron-level resolution by micro/nano processing technology and intelligent data analysis. As one of the most important sensor components in electronic skin (E-skin), tactile sensors have become a popular international research area due to their potential applications in wearable human health monitoring and care systems, advanced robotics, artificial intelligence, and human–machine interfaces. Among all kinds of tactile sensors, flexible, low-cost, conformal, portable and wearable real-time-monitoring functional electronic devices based on graphene and its derivatives should be generally concerned as the next generation of sensing devices for E-skin applications [1–5]. On one hand, the inherent characteristics of graphene and its derivatives, such as a large surface area and planar geometry, good electrical conductivity (ultrahigh mobility, ballistic transport, anomalous quantum Hall effect, nonzero minimum quantum conductivity, Anderson weak local change, and Klein tunneling), high chemical and thermal stabilities, and low toxicity, as well as being readily functionalizable, enable the effective detection of various stimuli [6–10]. On the other hand, additional unique superiorities, such as their lightweight, mechanical flexibility, and generally good processability, as well as their good compatibility with large-area and flexible solid supports, endow these materials with great potential for the manufacturing of sensing devices using a wide range of desirable or arbitrary solid supports [11–15]. Furthermore, diverse assembly and processing approaches, such as chemical modification, interfacial assembly, nanodoping, layer-by-layer assembly, laser scribing, dip-coating and others, can be employed to obtain graphene materials with new functions.

With a special emphasis on the state-of-the-art works published in 2016, these latest developments use the most advanced methods of material assembly, device construction and signal characterization and represent the forefront of graphene-based tactile sensors, laying the foundation and identifying the direction for future commercial applications. The main contents of this review provide a general synopsis on the functional supramolecular nanoassemblies of graphene and its derivatives with respect to progress during the tactile sensing era for E-skin applications. Some historically significant seminal works achieved before 2016, which are of paramount importance in shaping this field, are also highlighted to provide a foundation. For other interesting yet earlier studies, we encourage the interested readers to consult other excellent reports. This review is organized as follows: First, we briefly introduce the related concepts and preparation methods of graphene and its derivatives for tactile sensors. Then, with an emphasis on the impactful protocols of how to improve the performance of this kind of sensor, the unique roles and advantages of the employed graphene materials are discussed and highlighted by addressing representative paradigms. Finally, the current perspective and challenges of graphene sensors are outlined. We hope that the discussions will be beneficial to future investigations aimed at high-quality graphene-based tactile sensors.

## The Unique Roles and Advantages of Graphene Materials for Tactile Sensors

Since Geim and Novoselov discovered graphene in 2004, it has received tremendous attention as an ideal material to construct electronic devices due to its unique physical properties [16]. As is known, graphene, as the most celebrated of two-dimensional (2D) materials, possesses a unique *sp*^2^-hybridized crystal structure, where each carbon atom has three equivalent valence orbitals (one is an *s* orbital and the two others are *p* orbitals) together in a plane forming a triangle and a *p*_z_ orbital perpendicular to the basal plane, which arranges the carbon atoms in a honeycomb lattice. The hybridization of one *s* and two *p* orbitals leads to the formation of covalent *σ*-bonds with other neighboring carbons, and the *p*_z_ orbitals overlap each other to form delocalized *π*-bonds; the abundant delocalized electrons are responsible for the extraordinary electronic properties of graphene. The concentration of the electrons and holes of graphene can up to 1013 cm^−2^, and the mobility can reach 200,000 cm^2^ V^−1^ s^−1^ [17–20]. This property makes graphene an ideal active material in electronic devices.

Additionally, graphene has a perfect 2D structure, which offers abundant active sites on the basal plane to react with functional groups by means of conjugation reactions or the absorption of various functional moieties via hydrophobic interactions, dipole–dipole interactions, or *π*–*π* stacking [21]. This characteristic is the basic merit of graphene-based materials, from which we can build blocks of macroscopic graphene with novel structures and functionalities by means of self-assembly. From another point of view, most regular molecular assembly strategies make graphene the basic fundamental platform for external stimuli detection, as it can be modified by other functional nanomaterials [22–24]. Particularly, graphene oxide (GO) is an atomic-thick graphene fragment possessing hydroxyl and epoxide functional groups in the basal plane and carbonyl and carboxyl groups at the edges that express many distinguished advantages, such as facile preparation, mass production, chemical modification, interfacial activities, and low-cost. Reduced GO (RGO) not only is decorated with multiple oxygen-containing functional groups but also restores the good electronic, thermal, and mechanical properties of graphene. Therefore, abundant organic synthesis principles can be employed with functional supramolecular nanoassemblies of *π*-conjugated molecules to realize a responsiveness to various stimuli, confirming graphene materials to be excellent scaffolds for various sensors [25–30].

Moreover, graphene materials also possess excellent transmittance and mechanical properties with light transmittances of up to 97.7%, fracture strains of up to 25% and a Young’s modulus of ≈ 1.1 TPa, which provide graphene a significant opportunity for the construction of flexible and stretchable electronic devices used in tactile detection [31, 32].

Most importantly, many studies have discussed the physical properties of graphene and its sample preparation routes [33–35]. For example, (1) graphene deposited by mechanical exfoliation is commonly used for fundamental research due to the quality exhibiting near-inherent properties [36]; (2) chemical vapor deposition (CVD) and solution processing methods, which can scale up the production of graphene, are beneficial for the construction of tactile sensors based on graphene materials [37, 38]; and (3) laser scribing, plasma-enhanced CVD and spray-deposited graphene from solution are also effective ways to fabricate active materials for tactile detection and have attracted significant attention [39–41]. However, different assembly methods can possibly lead to clear differences in the fabricated graphene properties, which lead to different fundamental physics for strain sensors.

## How to Improve the Performance of Graphene-Based Tactile Sensors?

### Capacitive Tactile Sensors

In recent years, great progress has been made in fabricating pressure sensors based on different sensing mechanisms, including capacitive [42, 43], transistor-based [44, 45], piezoresistive [46, 47], triboelectric [48, 49], and optical sensing technologies [50]. Among the abovementioned protocols, touch-sensing devices based on the capacitive effect play an important role by taking the advantage of their inherent flexibility, low-power consumption, fast response speed, simple device structure, and low-cost scalable fabrication processes [7, 10]. Capacitance-type tactile sensors contain two conductive layers separated by an elastomer dielectric layer. The capacitance (*C*) of a parallel plate capacitor can be defined as *C* = *ε*_0_*εA*/*d*, where *ε*_0_, *ε*, *d*, and *A* are the vacuum dielectric constant, the relative permittivity of the elastomer dielectric, the distance, and the overlapped area between the two parallel plates, respectively. With excellent electrical properties, mechanical flexibility and optical transmittance, graphene has become one of the most promising materials for electrodes in tactile piezocapacitive sensors.

**Fig. 1:**
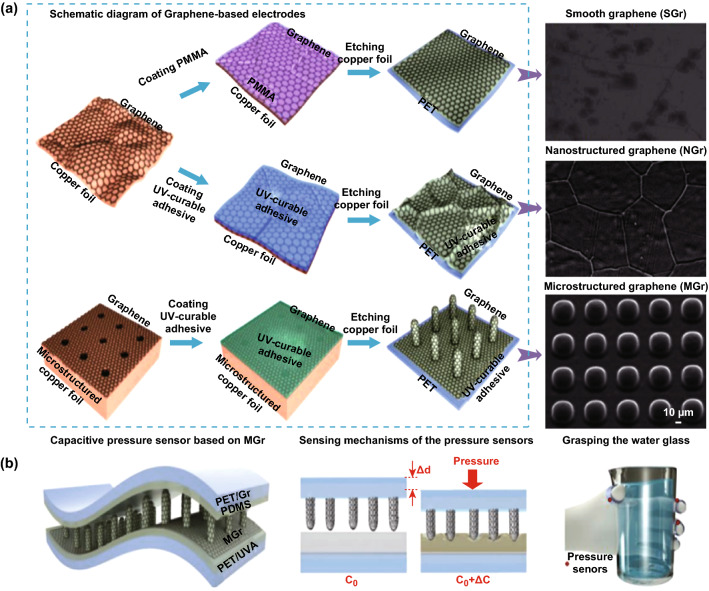
Typical capacitance-type tactile sensors with graphene as electrodes. **a** Schematic diagram of the fabrication processes for different conformal graphene electrodes and SEM images of three kinds of graphene films derived from PMMA-based, ultraviolet-curable adhesive-mediated, and microconformal transfer methods. **b** Illustration of a capacitive pressure sensor based on MGrE, a schematic diagram of the sensing mechanisms and grasping with the proposed pressure sensor. Reproduced with permission from Ref. [42]. Copyright 2019 American Chemical Society

For example, to obtain a tunable-sensitivity flexible pressure sensor, a classic method was employed by Luo and coworkers, wherein graphene was used as the electrodes, and polydimethylsiloxane (PDMS) pyramids with different spacings were used as the dielectric layer [42]. By a theoretical calculation model, the authors simulated the relationship curve between the sensitivity and PDMS pyramids with different spacings. The spacing of the pyramids was found to be a main factor affecting the sensitivity of the capacitance pressure sensor, and the measurement data were consistent with the simulation results. More importantly, with the help of graphene electrodes, pressure sensor devices with flexibility and reliability were achieved. Additionally, Yang and coworkers demonstrated a novel 3D microconformal graphene electrodes for ultrasensitive and tunable flexible capacitive pressure sensors, wherein smooth, nanostructured and microstructured flexible graphene electrodes (MGrE) were controllably fabricated via a PMMA-mediated transfer method, ultraviolet-curable adhesive-mediated transfer method, and microconformal transfer method, respectively (Fig. 1a). Owing to the roughness of the electrodes effectively improving the performance of capacitive tactile sensors and the tunable sensitivity via controllable microconformal structures, a capacitive pressure sensor with a high sensitivity, fast response speed, ultralow detection limit, tunable sensitivity, high flexibility, and high stability was obtained by sandwiching the PDMS dielectric layer between the top MGrE and bottom electrode, as shown in Fig. 1b. The as-fabricated MGrE-based tactile sensor could be used for monitoring blood pressure and sensing the capacitance response induced by droplets of water falling.

In the above configurations, the tunable sensitivity was obtained by changing the space between the pyramids or the morphology of graphene; indeed, the tunability of the suspended membrane area and the dielectric gap were the most important factors to the sensing performance (Fig. 1b). Although such graphene pressure sensors exhibit potential for application in wearable products such as E-skin (Fig. 1b), the size of the sensor cannot be decreased, resulting in a nonlinear pressure transduction and a limited dynamic operating range. Berger et al. [43] solved this issue by applying a novel strained membrane transfer and optimizing the sensor architecture. Pressure sensor devices with novel structures were fabricated by the following steps: (1) a chip with CVD graphene was coated with a layer of polymethylmethacrylate (PMMA) to form a graphene-polymer heterostructure membrane. (2) Then, the top surface was adhered to by a tape support window, which was lifted off the Si/SiO_2_ wafer by wet etching. (3) On another substrate, a piece of Si/SiO_2_ wafer was patterned by deep reactive ion etching to form an array of circular or hexagonal holes of a given diameter, periodicity and depth, arranged in various patterns such as a hexagonally packed lattice. (4) The graphene-polymer membrane was aligned with the patterned SiO_2_ surface using a tape-supported transfer process. It was found that sensors covering an area of just 1 mm^2^ showed reproducible pressure transduction under static and dynamic loading up to pressures of 250 kPa. The measured capacitance change in response to pressure was in good agreement with calculations.

The microstructure dielectric layer of the abovementioned devices to some extent can overcome the slow response and relaxation times caused by the high viscoelasticity of PDMS, leading to a substantially higher sensitivity and faster response/relaxation time. However, the construction of such capacitive sensors often requires intricate processes such as traditional lithography and *e*-beam evaporation, which are generally tedious to work and crosstalk between adjacent cells is inevitable. These drawbacks can be overcome simply by replacing the dielectric layer. Nylon netting composed of polyethylene terephthalate (PET) is a flexible, low-cost insulating polymer with a regular microporous structure and excellent mechanical properties, and was first selected and sandwiched between graphene films by He et al. [51] as the dielectric layer of a capacitive pressure sensor. Such devices have the advantages of excellent pressure-sensing sensitivity, ultralow detection limit, outstanding mechanical stability and ultrafast response speed, which enable the detection of fast variations in a small applied pressure from morphologically changing processes, e.g., the falling of a droplet onto the sensor. Moreover, a capacitive pressure sensor array was fabricated for demonstrating the ability to monitor spatial pressure distribution.

**Fig. 2:**
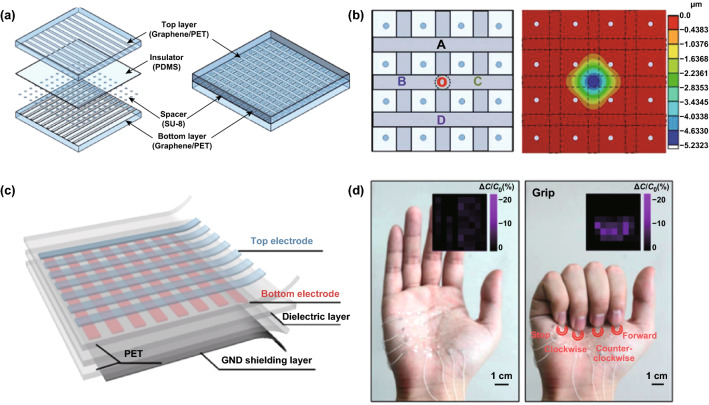
Typical crosstalk-free, multipoint recognition of flexible, and transparent capacitive graphene-based tactile sensors. **a** Schematic illustration of the sensor array composed of graphene-patterned top and bottom PET layers, PDMS insulator, and SU-8 spacers. **b** Schematic illustration of the 3 × 3 tactile cell array and the finite element analysis result for deflection of the top layer under 8 kPa applied to the center of cell-O. Reproduced with permission from Ref. [52]. Copyright 2017 WILEY–VCH Verlag GmbH & Co. KGaA, Weinheim. **c** Schematic diagram illustrating the concept of a graphene-based capacitive sensor. The sensor consists of three layers. The top and bottom layers are composed of patterned graphene electrodes on a PET film substrate. **d** Optical images of stretchable devices mounted on a palm for remote controlling the toy car. Inset of each image shows relative capacitance changes for spread (left) and grip (right) statuses of the hand. Reproduced with permission from Ref. [53]. Copyright 2017 American Chemical Society

An air gap between the surrounding spacers in each tactile cell is another effective method to reduce the difficulty in capacitive tactile sensor preparation. Pyo et al. [52] presented a capacitive tactile sensor comprised of monolayer graphene electrodes that were separated by spacers, which formed air gaps. As shown in Fig. 2a, the graphene electrodes were patterned and assembled on PET, while PDMS and SU-8 served as the dielectric and spacer between facing graphene electrodes, respectively. By utilizing the meritorious properties of graphene and the structural design of the air gap, the as-fabricated tactile sensor exhibited mechanical flexibility and an optical transparency in the visible range, along with a high pressure sensitivity (6.55% kPa^−1^), rapid response (≈ 70 ms), and high stability over 2500 cycles of loading/unloading. The authors also demonstrated a pixelated sensor array for pressure mapping without any significant crosstalk between adjacent cells, as shown in Fig. 2b.

From the above examples we can see that most of the capacitive tactile sensors have mainly focused on pressure or strain sensors that transduce physical touch into electronic signals, which cannot fulfill the demands of E-skin applications. Indeed, in addition to a position-sensing capability through contact, a 3D-sensing capability for the recognition of 3D shapes and the distance of approaching objects before contact occurs is significantly important both in wearable electronics applications and in the robotics field [53]. Furthermore, plausible mimics of multifunctional human skin will require multimodal detection, including temperature, humidity, and pressure, integrated into a single pixel [54]. To address these issues, a graphene-based touch sensor with an overall area of 4 × 6 cm^2^ and 8 × 8 array (64 channels) was fabricated by Kang and coworkers [53]. As displayed in Fig. 2c, this device was comprised of four main components. Ultrathin PET was used as the top and bottom substrates; triple-layer graphene, which was chemically doped with bis(trifluoromethane) sulfonamide (TFSA), was used as the transparent electrode; the acrylic polymer was used as the dielectric layers to separate top and bottom electrodes; and monolayer graphene was used as the shielding layer. By taking advantage of the unique properties of graphene and the thin device geometry, multitouch, spread, and scroll operation modes could be exhibited, and all remained stable, even on a curved forearm. As a result, this device can be integrated with highly deformable areas of the human body, including the forearms and palms, to sense both contact and noncontact modes, as shown in Fig. 2d.

**Fig. 3:**
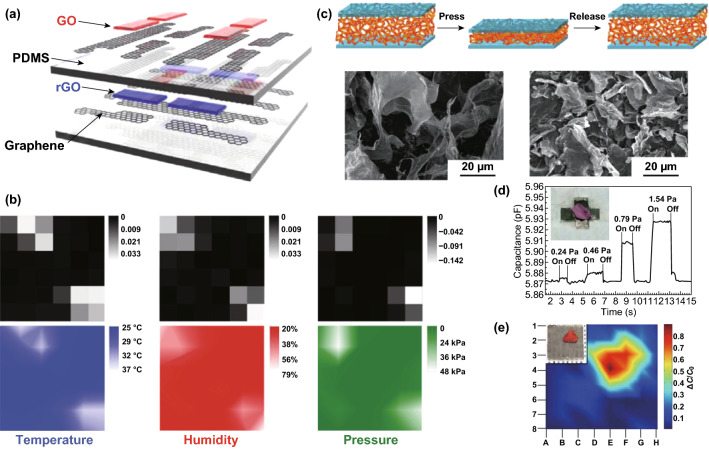
Versatile graphene and its derivatives are used to fabricate multifunctional capacitance-type tactile sensors. **a** Schematic diagram showing the four pixels (2 × 2) of the multimodal E-skin sensor, which were capable of mapping three individual stimuli including humidity, temperature and pressure. **b** Black-and-white maps of the calculated sensitivities of the three sensors during the finger pressing event (top), and a 2D color map of the distributions of the corresponding temperatures (blue), humidity (red), and pressures (green). Reproduced with permission from Ref. [54]. Copyright 2016 WILEY–VCH Verlag GmbH & Co. KGaA, Weinheim. **c** Schematic of the loading–unloading cycle for the pressure sensor with GO foam as a dielectric material. **d** Transient response to the placement and removal of several ultrasmall weights in the GO foam-based sensor. Inset: a petal on the sensor. **e** The pressure response to the strawberry. Insert: Bird’s eye view of the strawberry standing on the sensor arrays. Reproduced with permission from Ref. [56]. Copyright 2016 Elsevier Ltd

Graphene and its derivatives are versatile and sometimes can be used as a good dielectric material for capacitive pressure sensors. By adjusting the proportion of graphene and NH_4_HCO_3_ in a PDMS sponge, Kou and coworkers achieved a composite. When the sponge was sandwiched between two electrodes, a flexible wireless pressure sensor with a high sensitivity, wide operating rage, rapid response time, low detection limit, and good stability was obtained [55]. Ho et al. [54] developed a transparent and stretchable all-graphene multifunctional E-skin sensor matrix, wherein humidity, thermal, and pressure sensors were judiciously integrated into a layer-by-layer geometry through a simple lamination process. As shown in Fig. 3a, b, high-quality large-area CVD graphene was used to form the electrodes and interconnects for these three sensors, while GO and RGO were used as the active sensing materials for the humidity and temperature sensors, respectively. The 2D color maps of the simultaneous multifunctional sensing were collected without mutual interference of the electrical signals. Another fascinating all-graphene capacitive tactile sensor used for E-skin was fabricated by Wan and coworkers, wherein RGO was used as the electrodes, and GO foam, with excellent elastic property, was used as the dielectric material [56]. By utilizing the inherent insulating property of GO and the porous structure of the 3D GO sponge, the distance between the upper and the bottom electrodes decreased as an external pressure was applied; this decrease led to an increase in the capacitance, as shown in Fig. 3c, d. As a result, a tactile sensor with outstanding pressure sensitivity in a low-pressure regime was achieved, and prototype capacitive pressure sensor arrays of 8 × 8 pixels, with enough spatial resolution to detect the placement of a strawberry, were realized (Fig. 3e). Apart from, the GO sponge structure, a micropatterned graphene/PDMS composite was also employed as the dielectric layer [57]. With a wrinkled continuous Au pattern as an antenna and electrode and a folded PDMS cavity as the substrate, a flexible high-performance pressure sensor was obtained.

As seen from the above examples, both graphene layer and graphene foam can be used to construct high-performance capacitive tactile sensors. Graphene layers with good morphology, high crystallinity and uniform thickness are usually obtained through CVD. The graphene electrodes of capacitive tactile sensors obtained in this way not only present excellent electrical properties but also control the transparency through altering the growth process. Furthermore, the electrodes can also conform to substrates of different surface morphologies, thus, realizing the patterning of electrodes and ultimately improving the tactile sensitivity of devices. What is more interesting is that the CVD method can achieve large-area graphene layers, making the realization of integrated of tactile sensor components easier and laying a solid foundation for tactile sensors with good spatial resolution. However, the high energy consumption, high cost and high pollution stemming from the production process pose difficulties to realizing industrial production. In contrast, graphene foams are usually obtained through GO, which can be prepared in large quantities by solution methods at room temperature. Furthermore, GO has different electrical properties due to the varying degrees of reduction, which enables it to be used as both electrodes and the insulating layer of capacitance tactile sensors. When the graphene foam, which is porous and flexible, acts as the dielectric layer, the distance between the two parallel plates is easily adjusted under the action of external forces, thus, greatly improving the sensing performance of capacitive tactile sensors. However, even with a strong reduction, the oxygen-containing functional groups do not completely disappear, which greatly reduces the crystallinity and conductivity of RGO, and results in the material not being a good electrode for capacitive tactile sensors. Therefore, due to the graphene layer and the graphene foam having respective advantages and disadvantages for constructing capacitive tactile sensors, suitable forms of graphene and its derivatives with different morphologies should be chosen according to the actual practical applications.

### Piezoresistive Tactile Sensors

Although capacitive tactile sensors based on versatile graphene materials forge ahead on wearable electronic devices, it has been widely accepted that high sensitivity, high resolution, and mass production can hardly be realized at the same time, and the constructed devices are still far from practical uses. Owning to the excellent electrical property of graphene and its nanoscale flexibility, minor stress deformations could lead to a dramatic change in resistance [31]. Therefore, graphene-based piezoresistive sensors have become the most commonly used electromechanical sensors with relatively simple read-out systems and offer high flexibility and stretchability [58]. The mechanisms of graphene-based tactile piezoresistive sensors can be described by the following two types: due to the breaking of sublattice symmetry under uniaxial strain, the bandgap of graphene can be opened to increase its resistance; the fragments of a conductive network assembled by graphene and its derivatives can connect with each other under strain or pressure to change the resistance and recover when the external force is removed. In graphene-based piezoresistive sensors, the resistance of the graphene is defined as *R* = *ρL*/*A*, where ρ is the resistivity, *L* is the length, and *A* is the average cross-sectional area. When sensors are in operation, various related parameters are used to evaluate their qualities. Among them, the most fundamental parameter is the gauge factor (GF), which reflects the sensitivity to external physical action. The GF is defined as GF = (∆*R*/*R*)/*ε*, where Δ*R*/*R* is the normalized resistance and ε is the mechanical strain/pressure. A higher GF means a higher sensitivity [32]. To achieve a higher GF, various assembly methods have been applied in recent studies.

#### Graphene Tactile Sensors Using 1D Structures

**Fig. 4:**
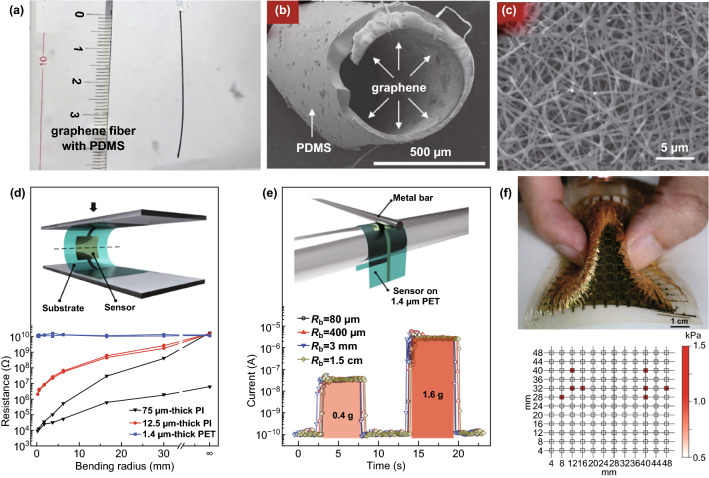
Typical 1D graphene architectures used for piezoresistive pressure sensors. **a** Photograph of a graphene fiber with a PDMS support. **b** SEM image of the cross-sectional view of a graphene/PDMS hollow tubing after Ni removal. Reproduced with permission from Ref. [46]. Copyright 2017 Elsevier Ltd. **c** FESEM image of the randomly stacked electrospun nanofibers. **d** Schematic showing the pressure response measurement of the sensor in bending and response curves when bent to a radius of 180 µm for different substrate thicknesses. **e** Tested pressure response of the device in the bent state and the response of the device fabricated on a 1.4-µm-thick PET substrate for bending radii from 15 to 80 µm for different normal forces. **f** Photograph of an integrated sensor array attached to the surface of a soft balloon, to which a pressure was applied by a pinching motion (top panel). Measured pressure data distribution under complex bending, showing no pressure signal from deformation, such as wrinkling (bottom panel). Reproduced with permission from Ref. [47]. Copyright 2016 Macmillan Publishers Limited

As the high aspect ratio of 1D architecture favors the rapid capture and release of external stimuli, increasing effort has been focused on fabricating pressure sensors based on 1D active material. However, the innate 2D structure of graphene makes obtaining 1D microscopic structure difficult. Thus, we need to search for other tools to help. As reported by Nakamura and coworkers, using a nickel wire as a template, 1D hollow tubing CVD graphene fibers (TGFs) could be obtained, coated with PDMS, and used as the active material for resistance-type strain sensors, as shown in Fig. 4a, b [46]. During the process of charge conduction, PDMS acted as a barrier in a bundle to bundle hopping, which made the TGF-based strain sensor possess better sensing properties than that of multiwall carbon nanotube (MWCNT)/PDMS composite-based strain sensors.

Electrostatic spinning is a special fiber manufacturing process in which a polymer solution or melt is sprayed in a strong electric field. By taking advantage of this commonly used method, composite nanofibers of carbon nanotubes and graphene were fabricated by Lee et al. [47] (as shown in Fig. 4c), wherein graphene was introduced to improve the pressure sensitivity. According to the authors’ simulation, these fibers changed their relative alignment to accommodate a bending deformation, thus, reducing the strain in individual fibers. Based on this fascinating result, extraordinarily small bending-sensitive, ultra flexible, and optically transparent resistive-type pressure sensors were fabricated. These sensors could be used to accurately evaluate external stimuli with curvilinear and dynamic surfaces; even when the sensors were bent to a radius as small as 80 µm, the sensor properties remained practically unchanged without bending interference, as shown in Fig. 4d, e. Furthermore, as shown in Fig. 4f, such a bending-insensitive device array could be used to accurately measure the distribution of the pressure normal to the soft movable 3D surface of a balloon that was being pressed by a soft object, such as a finger, without suffering from the inaccuracy induced by mechanical deformations, such as wrinkling and twisting. These excellent results lay a good foundation for the practical application of 1D graphene-based tactile sensors to E-skin.

GO and RGO contain abundant oxygen functional groups on their basal plane and edges, which make their self-assembly into 1D fiber architectures via solution processes possible. Fu and coworkers prepared a kind of conductive glass fibers (GFs) fabric by dip-coating GO on the surfaces of GFs, followed with an HI reducing process [59]. Taking advantage of the GFs with a high mechanical performance as a reinforcement filler and silicone resin with excellent flexibility as the matrix, the fabricated RGO@GFs/silicone composite simultaneously exhibited a high tensile strength and good flexibility. Yin et al. [60] dropped cellulose acetate fiber bundles into an as-prepared RGO aqueous solution to obtain synergetic fiber (SF)/RGO layers. When stretched, the fiber bundles fractured into gaps, islands, and bundles bridging the gaps; thus, the conductive fiber bundles could serve as mechanical sensors capable of detecting trace tensile strain down to 0.05% with a high sensitivity.

#### Graphene Tactile Sensors Using 2D Structures

**Fig. 5:**
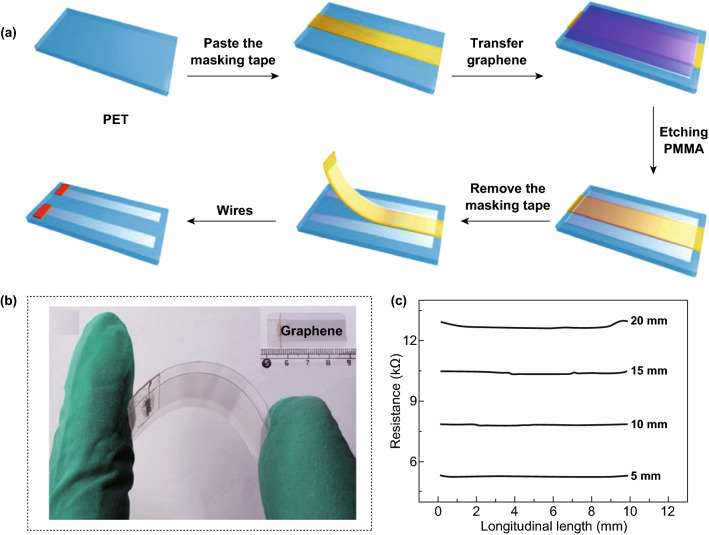
2D graphene films obtained by CVD used for piezoresistive pressure sensors. **a** Schematic illustration of the fabrication procedure of a tactile sensor composed of 2D graphene films and a PET substrate. Reproduced with permission from Ref. [62]. Copyright 2018 Springer Science + Business Media, LLC, part of Springer Nature. **b** Optical photograph of an ultrathin, transparent and flexible tactile sensor. Inset: The geometric dimension of the sensor. **c** Sensitivity of the device to longitudinal displacement at different axial distances of 5, 10, 15, and 20 mm. Reproduced with permission from Ref. [58]. Copyright 2017 The Royal Society of Chemistry

With the increasing demand for high-conductivity films, 2D graphene films have attracted significant attention due to their transparency and flexibility for wide-ranging application in optoelectronics, light-emitting diodes, solar cells, and sensors [11]. For tactile sensors used in E-skin, an abundance of facile synthetic methods for producing 2D graphene thin films exists. The CVD method is the most widely used approach to fabricate high-quality 2D graphene films, and many interesting works based on this method have been reported [59, 60], Recently, Li et al. [61] fabricated a tactile sensor based on a CVD graphene film-boron nitride (BN) heterostructure, wherein monolayer graphene was sandwiched between two layers of vertically stacked dielectric BN nanofilms. With the protection of the BN layers, the oxidation and contamination of graphene were effectively avoided. Xu et al. [62] constructed an ultrathin and flexible tactile sensing element based on few-layer CVD graphene films. As shown in Fig. 5a, the sensor was assembled through a very simple method consisting of a PET substrate and two unconnected graphene films. The excellent optical transparency made the sensor promising for a broad range of applications, including smart windows with a rainy weather warning. By means of a pair of compliant conductive plates, a novel tactile sensor, which could reflect the displacement of touch with sensitivity, excellent durability and fast response, was fabricated by Xu and coworkers (Fig. 5b); the plates were adhered to CVD graphene films, as the surface layer of a PET substrate, and a transparent elastic adhesive was sandwiched between the electrodes [58]. As the distance between a touch point and the electrode of the as-fabricated sensing device determined the change in resistance, this particular structure could reflect 1D touch, as shown in Fig. 5c. To realize the spatial resolution of a tactile sensor, a pressure sensor array with a 4 × 4 tactile sensing unit was constructed by Lv et al., and each sensing unit contained a polyimide (PI) substrate, CVD graphene/PET film and PDMS substrate bump [63]. The authors believed that the designed high-sensitivity flexible E-skin might have important application prospects in medical diagnosis, artificial intelligence, and other fields.

**Fig. 6:**
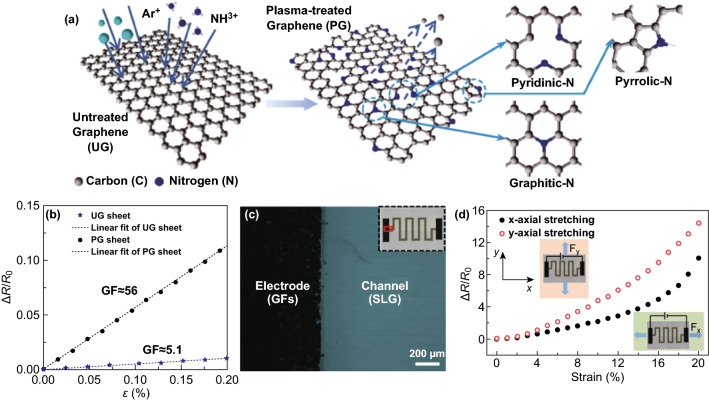
Modification and microstructure of CVD 2D graphene can further improve sensing performance of the devices. **a** Schematic representation of the plasma surface modification of a graphene sheet by NH_3_/Ar plasma. Nitrogen atoms are expected to substitute the carbon atoms in the form of pyridinic-N, pyrrolic-N, and graphitic-N configurations. **b** Relative change in resistance upon applied tensile strain for the fabricated sensors using the untreated graphene and plasma-treated graphene sheet. Reproduced with permission from Ref. [64]. Copyright 2017 American Chemical Society. **c** An optical image of the boundary between the single-layer graphene channel and the graphene flake thin-film electrode. Inset: Optical image of the entire device. **d** The resistance changes with tensile strain from 0 to 20%. The gauge factor is estimated at 42.2 for *x*-directional strain and 71.4 for *y*-directional strain with a rough linear fit. Insets show the schematics of force and measurement directions. Reproduced with permission from Ref. [65]. Copyright 2017 The Elsevier Ltd

In addition to directly using CVD graphene as an active material in tactile sensors, the modification and microstructure of graphene can further improve the sensing performance of devices. From the point of view of material modification, Haniff et al. [64] found that the straightforward NH_3_/Ar plasma treatment of graphene, changed its morphology, structure, chemical composition, and electrical properties. Due to the tunneling behavior originating at localized defects, the graphene structure doped with nitrogen atoms exhibited a significant increase in sensitivity by one order of magnitude compared to that of the unmodified graphene sheet, as shown in Fig. 6a, b. The integration of a serpentine-shaped pattern for single-layer graphene was another efficient way to improve the performance of graphene-based tactile sensors (Fig. 6c) [65]. Owing to the unique microstructure, the sensor was capable of stretching up to 20% with a high GF up to 42.2 and could provide functional extensions to bidirectional responses (Fig. 6d). In terms of the microstructure of the 2D material, there are no exactly flat graphene materials when the length in one dimension exceeds 10 nm [66]. It is appealing that compared with their flat counterparts, wrinkled structures could induce many novel physical properties and have several distinguishing application trends in the E-skin field [67]. Therefore, in recent years, great efforts have been made to seek methods for generating highly controlled wrinkling in graphene materials. Chen and coworkers reported a high-sensitivity, ultrathin, and transparent pressure sensor based on wrinkled graphene prepared by a facile liquid-phase shrink method [68]. A porous anodic aluminum oxide (AAO) membrane, with a thickness of only 200 nm, was used to isolate the two layers of graphene. When an external compression was applied to the as-fabricated device, the distance between two graphene wrinkles was changed to form current pathways. As a result, an ultrahigh operating sensitivity (up to 6.92 kPa^−1^) was obtained, substantially higher than that of tactile sensor devices with relatively flat graphene electrodes. More interestingly, as the complete separation of the two graphene layers occurred when the sensor was not subjected to any pressure, such a device could be used as an on/off and energy-saving device.

**Fig. 7:**
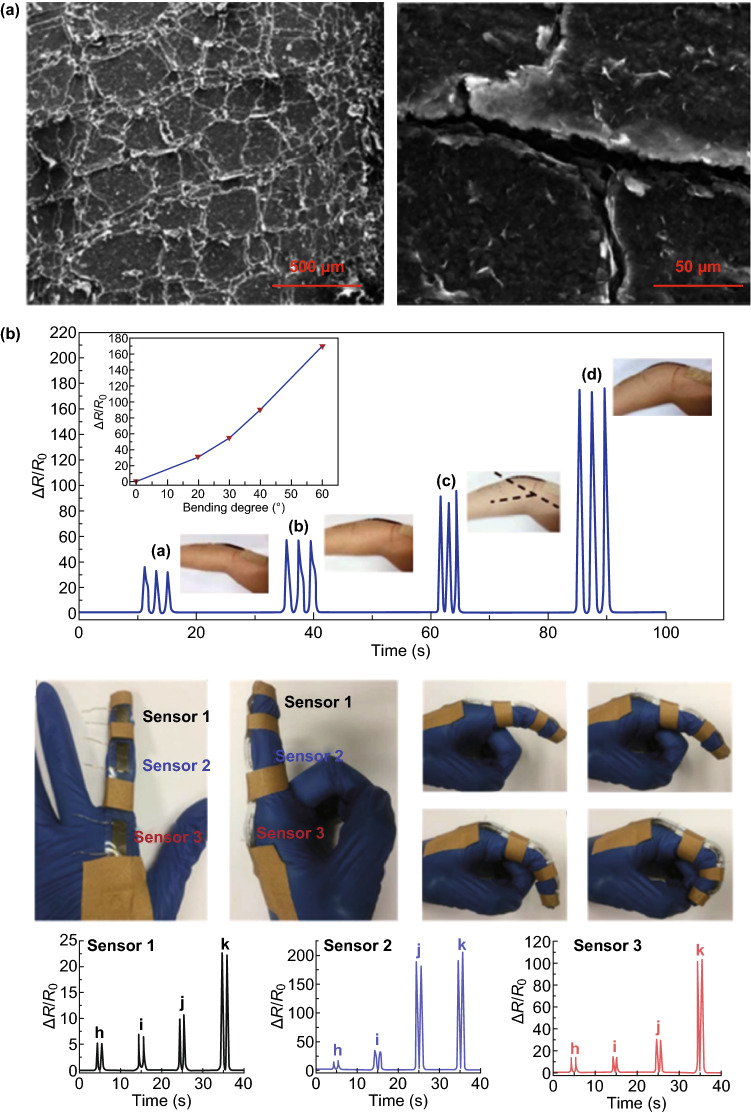
2D graphene films obtained by a solution fabrication process and used for piezoresistive pressure sensors. **a** The generated and enlarged scaled cracks under minor strain (left). The magnified SEM image of generated and enlarged scaled cracks under minor strain (right). **b** The strain sensor can meet the demands of subtle, large and complex human motion monitoring. Reproduced with permission from Ref. [69]. Copyright 2018 The Royal Society of Chemistry

Although CVD graphene-based tactile sensors show the high sensitivity and reliability needed for sensor devices, their low yield, high production costs, and complex processes hinder their development toward practical applications [15]. Fortunately, the functional diversity of graphene and its derivatives make solution processing possible and can provide environmentally friendly, low-cost, and scalable methods for the production of large-area ultrathin 2D graphene films [69, 70]. Yang and coworkers proposed an ultrasensitive strain sensor with a large strain range and ultrahigh GF (up to 1054) based on graphene armor scales by a simple solution fabrication process [69]. To achieve the graphene armor scales, graphene ink was sprayed uniformly on the surface of a PDMS substrate to form a 2D film. After wiring with copper wire and silver ink, another PDMS layer was used to encapsulate the whole structure. Then, the sensor was stretched and recovered within a range of 50% ten times to generate the graphene armor scales, as shown in Fig. 7a. Due to the excellent performance, this strain sensor could meet the demands of E-skin for subtle, large and complex human motion monitoring (Fig. 7b) and indicated tremendous application potential for health monitoring, mechanical control, real-time motion monitoring and more. Another relevant example in terms of solution processing methods was presented by Zhang et al, wherein RGO was coated on micropyramid PDMS arrays via layer-by-layer assembly [70]. This feature size would be easily integrated into a cell array with sufficient spatial resolution and constructed signal collection. Additionally, by employing an in situ chemical reduction method with the eco-friendly reducing agent vitamin C, a free-standing graphene film presented surface fluctuations, and a fluffy, layered structure was obtained in the cross section [71]. Owing to this advanced structure, a pressure sensor based on such a graphene film displayed a high sensitivity along with an extraordinarily ultra-wide operation range. Cost-effective methods, such as direct laser scribing PDMS and direct laser reduction of GO, can also help us to obtain high-performance tactile sensors [62, 72].

#### Graphene Tactile Sensors Using 3D Porous Structures

Active materials based on a 3D porous structure are the most common well-shaped and self-supported graphene hierarchical nanostructures used in graphene-based tactile sensors. Based on the large stacking interfaces and the *π*–*π* interface interactions between graphene sheets, those 3D materials with an ultralight density and flexibility also possess a high conductivity and mechanical strength. In addition, their scalable production makes for an attractive choice for practical implementation [21]. Recently, researchers have developed several porous materials as templates to generate 3D graphene porous structures, such as polymer sponges (including polyurethane (PU) and polyvinyl chloride (PVC)) [73–78], various fabrics [79–81], cellulose paper [82], multilayer silk [83], all kinds of metal foams [84–86], and others [87–89].

**Fig. 8:**
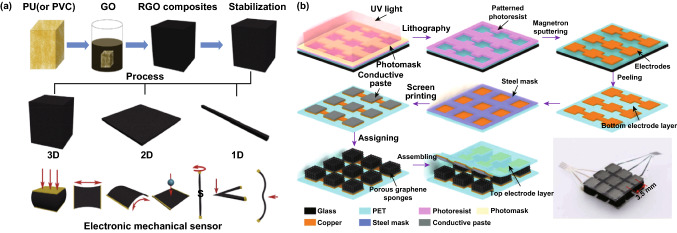
Schematic of the fabrication procedures of a 3D graphene material with a sponge as a template. **a** Preparation route for RGO/PU (or PVC) sponges and their multidimensional sensor applications. Reproduced with permission from Ref. [74]. Copyright 2017 Elsevier Ltd. **b** The fabrication procedures of the flexible tactile sensor array using PU as a template. Reproduced with permission from Ref. [76]. Copyright 2018 IEEE

**Fig. 9:**
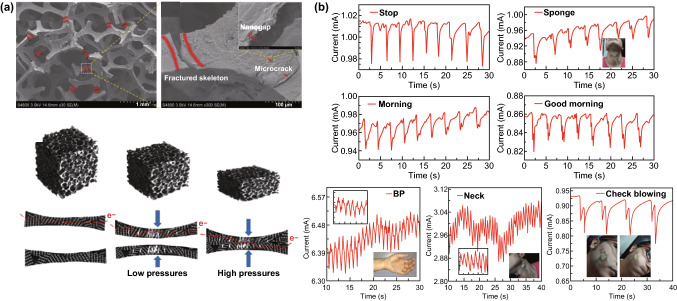
The mechanism and application of 3D graphene materials with sponges as template-based tactile sensors. **a** SEM images of the MWCNT/RGO@PU-5C sponges with nanogaps, microcracks and fractured skeletons (The top panel). Schematic of the MWNT-RGO flake-wrapped PU foam with the magnified image of its individual skeleton at three stages, without pressure, at low pressure, and at high pressure (The bottom panel). Reproduced with permission from Ref. [77]. Copyright 2018 The Royal Society of Chemistry. **b** Real-time response of the MWNT-RGO@PU piezoresistive sensor for various small-scale motion monitoring applications was studied using the throat while pronouncing different words. Reproduced with permission from Ref. [73]. Copyright 2017 Wiley–VCH Verlag GmbH & Co. KGaA

A typical example using commercially available PU and PVC sponges as templates was reported by Zhang et al., wherein graphene-wrapped sponges were obtained by soaking sponges in a hydroquinone/GO mixed solution and then vacuum annealing under certain conditions [74]. The as-constructed composites could be processed into different dimensions and differently shaped sensors to detect multiple forms of mechanical deformation, such as tensile strain, impact, bending, vibration, and twisting, as shown in Fig. 8a. By using a similar approach, Zhu and coworker fabricated graphene sponges via a dip-coating process that stacked graphene layers onto polyimide scaffolds in a homogeneous graphene solution with GO serving as the dispersant [76]. Then, as illustrated in Fig. 8b, a tactile sensor with 3 × 3 graphene sponge sensing units was constructed through photoetching, magnetron sputtering, and screen-printing processes. The authors’ believed that such a tactile sensor had potential for E-skin applications, such as monitoring body motion and other biomedical applications. Conductive PU sponges coated with synergistic MWCNTs and graphene prepared by solution methods could be used to construct more advanced tactile sensing devices by taking advantage of the synergistic effect from multiple mechanisms [73, 77]. As shown in Fig. 9a, under a low compression strain, MWNT-RGO@PU assumed nanogaps, microcracks and a fractured skeleton while at the stage of the “disconnect-connect” transition; whereas a high compression strain led to the compressive contact stage, where a conductive skeleton was displayed. The versatility of these sensors has been demonstrated in a wide range of E-skin applications, such as speech recognition, health monitoring, and body motion detection, as displayed in Fig. 9b. In addition to MWNTs, a conducting polymer such as polyaniline, with a large surface area and excellent electrical conductivity, can also be mixed with graphene to construct stretchable electronic devices to improve the sensing performance [75].

As is known, the majority of sponges provided on the market are PU sponges. The production process for this kind of chemical product is neither environmentally friendly nor conducive to human health, so tactile sensors based on PU sponges are difficult to realize for practical applications of E-skin [81, 83]. To achieve a similar functionality without the abovementioned environmental and health issues, various natural fabrics have been used to replace PU sponges. Liu and coworkers utilized silk as a support body to fabricate a 3D graphene structure, a graphene-silk pressure sensor with high sensitivity, good repeatability, flexibility, and comfort for skin was obtained [83]. Yuan et al. developed a facile, cost-effective, and scalable method for the fabrication of high-performance strain sensors based on a graphene-coated spring-like mesh network. Owing to the unique 3D structure of the spring-like mesh network, the tactile sensor could be used to detect various deformations, such as pressing, stretching, bending, and even subtle vibrations [81]. Mi and coworkers chose highly elastic fabric fibers as the functional carrier and then simply coated RGO on the fibers by plasma treatment, dip-coating and hydrothermal reduction steps, finally making a wearable strain sensor [83]. Lu and coworkers used low-cost, commercial 3D polyester nonwoven fabrics as scaffolds to construct highly sensitive wearable piezoresistive pressure sensors [90]. Kim collaborated with colleagues and fabricated RGO/SWCNT hybrid fabric-based strain-pressure sensors using a simple solution process. The RGO/SWCNT fabric sensor not only showed particularly high mechanical stability and flexibility during 100,000 bending tests but also exhibited excellent water-resistance properties after ten washing tests [79]. The superior sensing performances and economic fabrication processes belonging to these kinds of wearable tactile sensors have strengthened our confidence in smart clothing, which can be practical for applications in household, health-care, entertainment and robotics fields.

**Fig. 10:**
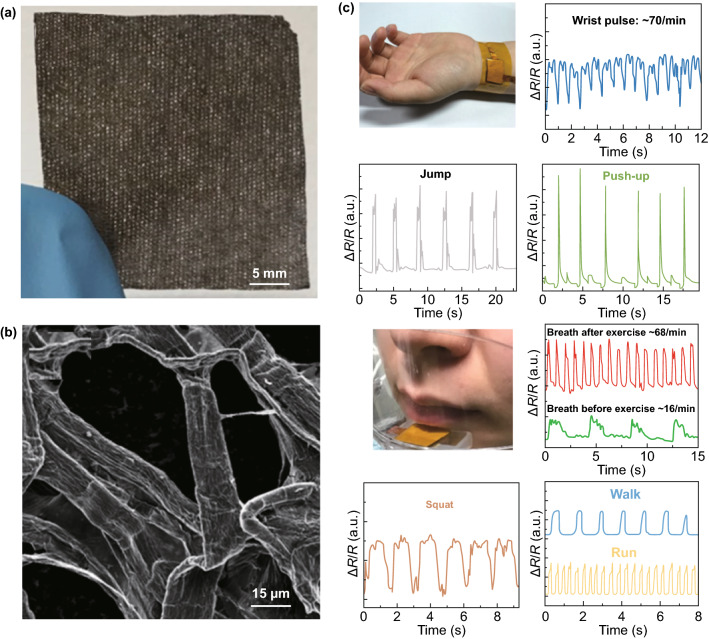
Typical paradigms for 3D graphene materials with paper as the template used for tactile sensing. **a** Tissue paper with RGO. **b** SEM photo of the tissue paper sensor at high magnification. **c** Applications for various intense motion detections. Reproduced with permission from Ref. [78]. Copyright 2017 American Chemical Society

In addition to natural fabrics, paper is another kind of environmentally friendly substrate with a 3D hierarchical nanostructure and good elasticity that also has potential to be a good alternative to improve the performance of pressure sensors. A typical paradigm was reported by Tao et al. [78], who mixed multilayer tissue papers with a GO solution to obtain GO paper; then, after an annealing process and the drawing out of a wire, a graphene-paper-based pressure sensor was constructed. The 3D structure of the tissue paper with RGO is shown in Fig. 10a, b; sensors applied in pulse detection, respiratory detection, and voice recognition, as well as the detection of various intensities of motion, are demonstrated in Fig. 10c. Compared to most reported graphene pressure sensors, this sensor realized the optimization of sensitivity and working range, which was especially suitable for wearable applications. The authors believed that this graphene-paper pressure sensor would have great potential in E-skin devices to achieve health monitoring and motion detection.

**Fig. 11:**
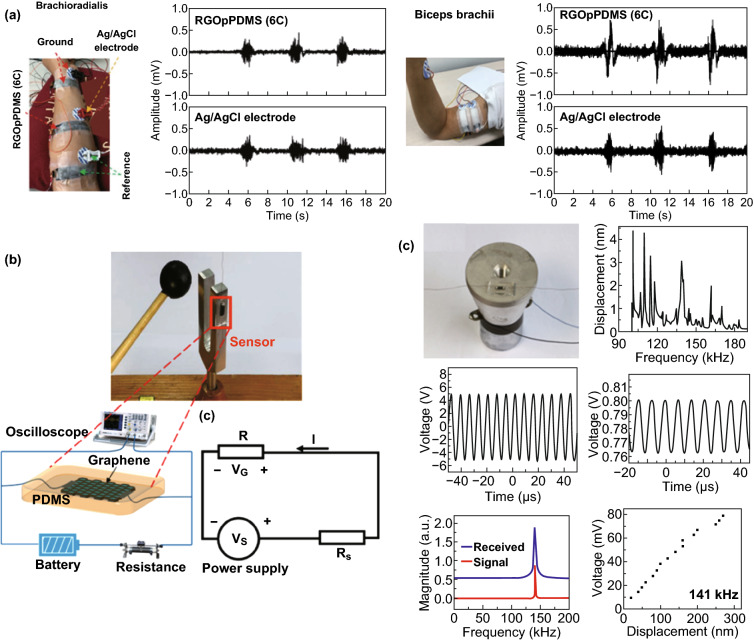
3D graphene materials with other polymer porous materials as templates used for tactile sensing. **a** Photograph of both electrodes on a brachioradialis muscle and on biceps brachii muscle for electromyogram measurement. Reproduced with permission from Ref. [88]. Copyright 2017 WILEY–VCH Verlag GmbH & Co. KGaA, Weinheim. **b** Experimental setup of the tuning fork vibration test and a schematic of the test circuit for the graphene film/PDMS sensor. **c** Photo of the piezoelectric ceramic transducer test. Reproduced with permission from Ref. [92]. Copyright 2017 AIP Publishing

Although the 3D graphene structures prepared through the abovementioned templates can significantly improve the sensing performance of tactile sensors, the opacity of sponges, fabrics and tissue paper hinders the construction of transparent E-skin [91, 92]. PDMS, an intrinsically elastic and extensible material with a high transparency, responds readily to tensile, torsional, and compression forces and has been widely used as a flexible substrate for various tactile sensors. Yun and coworkers employed simple coatings and a direct patterning method to fabricate RGO-sheet-wrapped PDMS porous conductive materials, without any complicated microfabrication processes [88]. Taking advantage of the inherent properties of PDMS and the high conductivity of RGO, the strain sensor exhibited a high sensitivity with a wide sensing range, which could be used to monitor large-scale human motion, as shown in Fig. 11a. The as-constructed graphene/PDMS porous structure could also be used for bioelectrodes to detect human electrophysiological signals. Encapsulating 3D graphene foam with PDMS is an alternative approach to obtain pressure sensors based on 3D porous graphene foam/PDMS [89–92]. By unidirectional freeze-drying and simple mechanical compression, a tactile sensor with excellent flexibility, high stretchability and sensing sensitivity, and anisotropic mechanical properties was fabricated by Zeng and coworkers [89]. Rinaldi et al. [81] found that the piezoresistive properties could be adjusted by varying the amount of graphene in the graphene/PDMS foams. Due to static mechanical forces or KHz vibration, the electronic band structure would become modified, leading to a significant resistance change in graphene. Based on this phenomenon, Zhang and coworkers found that the 3D graphene foam/PDMS could be used to detect frequency signals by both tuning fork tests and piezoelectric ceramic transducer tests, which showed a clear linear response from audio frequencies, including frequencies up to 141 kHz (the ultrasound range), as shown in Fig. 11b, c [92]. Zheng et al. employed such a facile approach to design highly stretchable graphene foam/PDMS composite films with tunable sensitivities and switching capabilities by simply controlling the thickness of the graphene foam [91]. Based on a 3D printing technique, Wang et al. successfully fabricated graphene/PDMS composites with long-range ordered porous structures. The resultant composites presented tunable and high gauge factors, along with excellent durability [93].

Beyond PDMS, other flexible porous polymers, such as porous inverse opal acetylcellulose (IOAC) films, thermoplastic polyurethane electrospun fibrous mats, poly(diallyldimethylammonium chloride), polyester textiles, and polyaniline (PANI) can also be used as templates to construct 3D graphene structures [89–97]. The special hierarchical conductive network endows 3D graphene-based tactile sensors with a good stretchability and high sensitivity. The nanoscaled PANI arrays greatly enhanced the strength and electrical conductivity of the 3D microarchitectural RGO sponge, endowing the pressure sensor with a high sensitivity, wide range and reliable sensing, a rapid response time, and excellent stability. Simultaneous, a porous IOAC film could be used not only as flexible microstructured substrates for highly sensitive motion sensing but also for the collection and analysis of ion concentrations in sweat by monitoring simple colorimetric changes or reflection-peak shifts, which resulted in this material having great application potential in the field of E-skin.

**Fig. 12:**
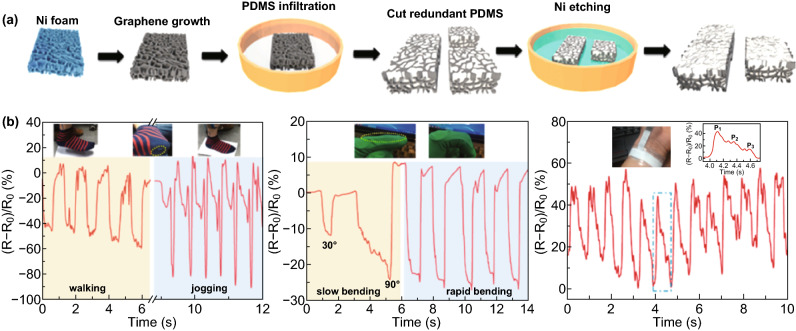
3D graphene materials with metal porous materials as templates used for tactile sensing. **a** Schematic process for fabricating pressure and strain sensors with the graphene porous network structure. **b** Signal variations in relative resistance corresponding to different walking/bending states and wrist blood pressure. Reproduced with permission from Ref. [85]. Copyright 2016 American Chemical Society

Flexible and transparent polymers to some extent can solve the problem of sensor transparency. Nevertheless, all of the porous structure cannot effectively remain in sensors after the polymer has infiltrated into the as-prepared graphene foam, and the pores can achieve enhanced sensing performances [97, 98]. To conquer this challenge, Pang and coworkers used nickel foam as a template and a chemical etching method to create a graphene porous network (GPN), as shown in Fig. 12a; this represents the first work of in situ GPN prepared in a polymer and used for pressure and strain applications [85]. Because of the pores in the GPN, the composite, as pressure and strain sensors, exhibited a wide pressure-sensing range and the highest sensitivity among graphene foam-based sensors, respectively, and could be used as E-skin to monitor or even recognize walking stages, finger bending degrees, and wrist blood pressure (Fig. 12b). Kim et al. [98] demonstrated a strain-pressure sensor with a high sensitivity and durability by combining molybdenum disulfide (MoS_2_) and Ecoflex with such a GPN. It was found that the conformal nanostructure of MoS_2_ on the GPN surface could produce improved resistance variations against external strain and pressure. As a result, the MoS_2_/GPN/Ecoflex sensor exhibited noticeably improved sensitivity over that of previously reported GPN/PDMS sensors in a pressure test. Copper foil, as the most commonly used metal for the preparation of graphene by CVD, has also been used to fabricate porous graphene 3D structures [75, 84].

Generally, the size and distribution of the pores in the foam, as well as the thickness of the pore walls, are essential to the sensitivity of graphene foam-based pressure sensors [99]. Therefore, developing new approaches for preparing graphene foams with satisfactory disorder to fabricate high-performance pressure sensors with acceptable sensitivities, detection limits, response times, and stabilities is significantly important. Due to the consistency of commercial production patterns and methods, the abovementioned templates are difficult to realize because of the highly disordered distributions of pore diameter and pore-wall thickness [100, 101]. Furthermore, to some extent, the scaffold materials increase not only the complexity of the sensor structure but also the weight of sensing devices [100]. Zang and coworkers introduced an ultrasonic dispersion method to solve this problem, and the porous structure was maintained by the freeze-drying process [101]. Due to the maintenance of the highly disordered structure of the ultrasonically dispersed GO before the freezing process, the RGOF sensors demonstrated an ultrahigh sensitivity of 22.8 kPa^−1^, an ultralow detection limit of approximately 0.1 Pa, and a superior separation of 0.2-pascal-scale difference.

**Fig. 13:**
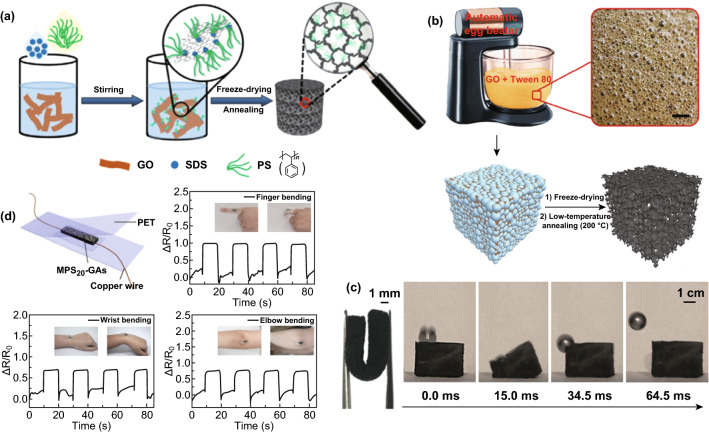
Typical 3D graphene structures via surfactant-assisted self-assembly used for tactile sensors. **a** Schematic illustration of the fabrication process of 3D microporous polystyrene/graphene aerogels. Reproduced with permission from Ref. [103]. Copyright 2016 Wiley–VCH Verlag GmbH & Co. KGaA, Weinheim. **b** Illustrations of the preparation of a sparkling graphene block. **c** Photograph of a sparkling graphene block bent to 180° (left) and real-time images from a high-speed camera showing that the sparkling graphene block can rapidly bounce a steel ball. Reproduced with permission from Ref. [102]. Copyright 2017 American Chemical Society. **d** The as-fabricated tactile sensor as a promising candidate for wearable devices. Reproduced with permission from Ref. [103]. Copyright 2016 Wiley–VCH Verlag GmbH & Co. KGaA, Weinheim

To fabricate graphene aerogels with ultralight, superelastic, and excellent mechanical and multifunctional properties, surfactants and crosslinkers are often employed in the synthesis system [100–102]. Qu’s group fabricated macroporous polystyrene/graphene aerogels (MPS-GAs) with the help of sodium dodecyl sulfate (SDS) by using a simple physical strategy in an aqueous emulsion containing polystyrene (PS) as a mediator [103]. The synthesis process is shown in Fig. 13a: (1) Cyclohexane containing PS was introduced into GO suspensions, followed by adding SDS into the system through vigorous stirring, in which SDS acted as a surfactant to decrease the surface tension and facilitate the stable and uniform dispersion of PS in the GO aqueous suspensions. (2) During this process, PS molecules, with a conjugated structure, could crosslink well with graphene sheets through *π*–*π* interactions. Then, the formed emulsions were immediately immersed into liquid nitrogen for 10 min to keep their macroporous structure. (3) The aerogels were obtained after lyophilization and a thermal treatment. Thereafter, the authors used polyethylene glycol sorbitol monooleate (Tween 80), instead of SDS, as a sparkling agent, and an automatic egg beater, instead of a blender, to obtain a sparkling graphene block (SGB) with bubbled cavities maintained well, as illustrated in Fig. 13b [102]. The 3D microporous graphene aerogels obtained by freeze-directed assembly and assisted by surfactants exhibited an excellent elasticity, even at 95% compressive strain, and could rebound a steel ball with an ultrafast recovery speed (~ 1085 mm s^−1^), making this material a promising candidate for applications in actuators, elastic conductors, strain/pressure sensors, and wearable devices, as shown in Fig. 13c, d. Xiao et al. developed a silane-crosslinked and modified graphene aerogel (SGA) using a novel and simple method involving the CVD of methyltriethoxysilane into a graphene oxide aerogel, wherein the compression recoverability could extend to 99.5% [100]. In addition to a high-tactile sensing performance, the compressible and ultralight structure could also serve as a fast and recyclable superadsorbent being able to adsorb various organic liquids with an ultrahigh capacity. Researchers also found that the addition of functional inorganic semiconductor materials, such as SnO_2_, GaN, and CdS, to the graphene aerogel 3D structure could enhance the tactile sensing properties, where the piezoresistive response was considerably higher than that of the bare aerogel [104, 105].

**Fig. 14:**
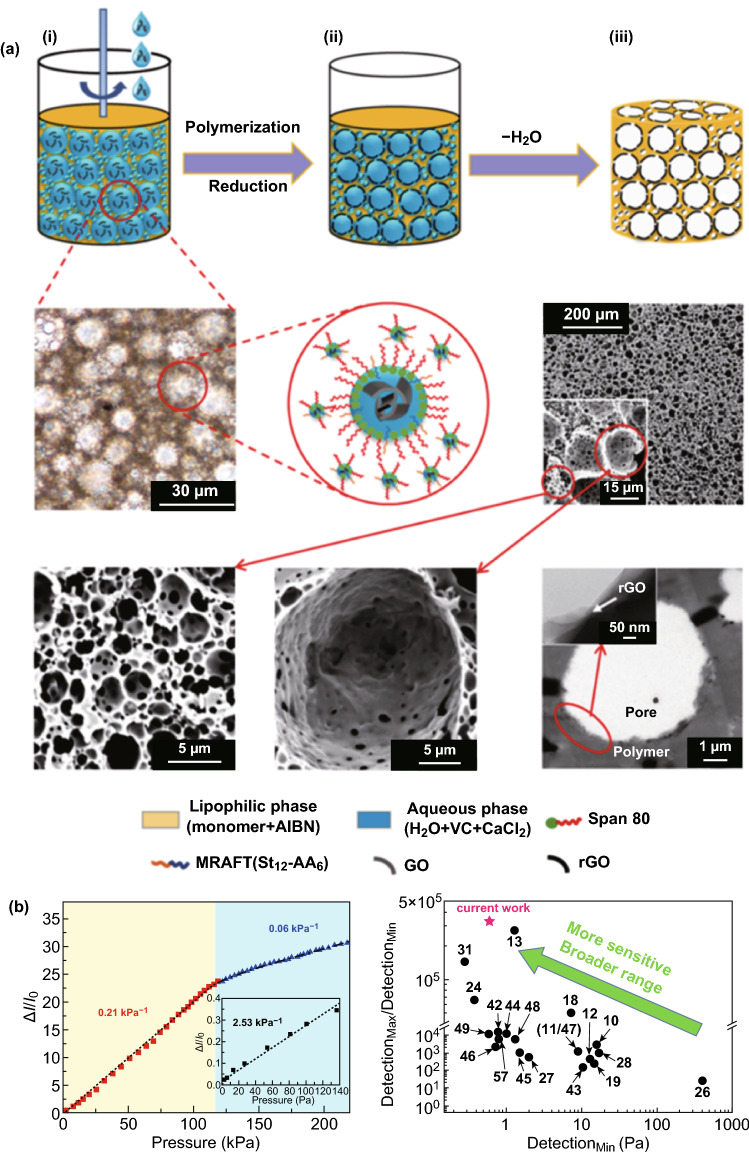
A novel method to obtain 3D graphene structures by using high-internal-phase emulsion (HIPE) as a template. **a** Schematic illustration of the procedure for fabricating the RGO@PolyHIPE foams via HIPE polymerization, optical microscopy images, and SEM images of the foam. **b** Relative change in the sensor’s current and pressure curves. The inset shows the relative current change in a small pressure range below 140 Pa (left). Comparison of the detection limit of minimum pressure and the responsive pressure range between the sensor described in the current work and previously reported sensors (right). Reproduced with permission from Ref. [106]. Copyright 2019 American Chemical Society

From the abovementioned 3D graphene-based tactile sensing paradigms for E-skin applications, we can see that most of the developed strategies focused on high sensitivity, while sensors capable of combing high sensitivities and broad dynamic ranges have barely been proposed. This inequality is because such materials are prone to saturation responses when attempting to obtain measurements involving high pressures [106]. By means of using a high-internal-phase emulsion (HIPE) as a template, a highly porous graphene material consisting of small pores packed between larger ones was fabricated by Yang and coworkers, wherein the inner walls were lined with RGO [106]. The procedure for fabricating RGO@PolyHIPE foams and the image of this kind of material are illustrated in Fig. 14a. Owing to the unique 3D hierarchical structure, the piezoresistive pressure sensor based on RGO@PolyHIPE foam was capable of a high sensitivity over a pressure range spanning from a mosquito touching the surface to an elephant standing on the surface, as shown in Fig. 14b. Tsui et al. reported piezoresistive responses from aerogels of graphene-coated SWCNTs, made using a facile and versatile sol–gel method [107]. With the synergistic effect of graphene and SWCNTs, the piezoresistivity of these aerogels spanned wide compressive pressures up to at least 120 kPa with sensitivity, and the piezoresistive responses did not show any creep for at least 1 h and 80 kPa of compressive static loading. Such sensing regimes allow tactile sensors based on 3D graphene structures to move closer to the practical application of E-skin.

#### Graphene Tactile Sensors Draw Inspiration from Nature

As we all know, many well-adapted hierarchical structures have been developed through natural selection and are critical for the survival of organisms. For example, to climb a vertical wall, the feet of the gecko have developed a kind of special hierarchical structure so that maximized contact area and intermolecular interactions could be realized [108], the self-cleaning ability of a lotus leaf mainly depends on the hydrophobic hierarchical structure of its surface [109], and the epidermal ridges on the surface of the human skin help us perceive the world. These typical examples have inspired us to design biomimetic materials for the fabrication of tactile sensors used in E-skin applications [110–112].

**Fig. 15:**
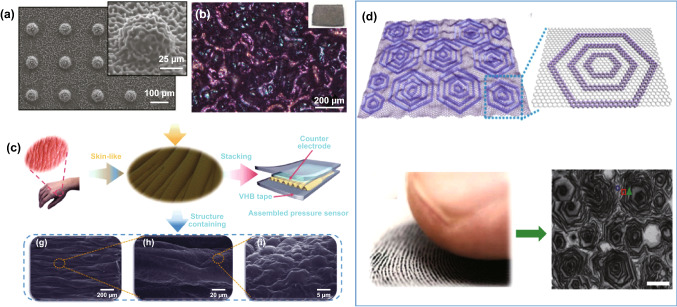
Bioinspired hierarchical graphene structures as active layers for pressure detection. **a** SEM image of hierarchically structured graphene/PDMS array. Inset is a magnified image of an individual structure. Platinum sputtering was omitted prior to the SEM imaging; a clear SEM image implies that the sample surface was fully covered with conducting graphene. Reproduced with permission from Ref. [109]. Copyright 2016 WILEY–VCH Verlag GmbH & Co. KGaA, Weinheim. **b** Photographs (insets) and optical micrographs of the RGO coating on PDMS after exposure to a high temperature. Reproduced with permission from Ref. [111]. Copyright 2018 American Chemical Society. **c** Picture of a human hand and partial enlargement (inset), and the schematic diagram of sandwich ultrasensitive pressure sensors based on the skin-like wrinkle film (top panel). SEM images showing the morphology of a skin-like wrinkle film (bottom panel). Reproduced with permission from Ref. [112]. Copyright 2018 The Royal Society of Chemistry. **d** Schematic illustration showing the structure of the 3D graphene film containing a continuous graphene film and closely packed concentric hexagonal graphene nanoribbon rings. Picture of a fingertip and its fingerprint. In addition, SEM image of a 3D graphene film on a SiO_2_/Si substrate. Reproduced with permission from Ref. [113]. Copyright 2018 Springer

By using a bioinspired hierarchical structure based on the surfaces of organs and consisting of PDMS covered with monolayer graphene (Fig. 15a), Bae and coworkers presented a high-performance piezoresistive pressure sensor device with a linear relationship between the applied pressure and output and with a high sensitivity over a wide range of pressures, specifically between 0 and 12 kPa [109]. Inspired by the skin’s epidermis, with high-performance force sensing, Pang et al. proposed a special surface morphology with a spinosum microstructure of random distribution via the combination of an abrasive paper template and RGO, as shown in Fig. 15b [111]. By taking advantage of the random distribution of the spinosum microstructure, the sensitivity of the graphene pressure sensor could reach 25.1 kPa^−1^ over a wide linear range of 0-2.6 kPa. As shown in Fig. 15c, following the same inspiration, a novel pressure sensor with a hierarchical structure and gradient RGO wrinkles was reported by Jia et al. [112]. The researchers found that benefiting from the skin-like structures, the pressure sensor demonstrated an outstanding sensitivity.

Except for human epidermis, the epidermal ridges on the skin of the human fingertip, which serve to amplify subtle external stimulations, can also inspire us to design highly sensitive fingertip skin-like pressure sensors. As shown in Fig. 15d, the growth of a 3D graphene film mimicking the morphology of fingertip skin via CVD was reported by Xia and coworkers [113]. The hierarchical structure of graphene and the PDMS films molded from a natural leaf contributed to the superior performance of the pressure sensor. Chun and coworkers reported that by introducing microstructures inspired by human fingerprints, a surface texture was successfully defined through fast Fourier transform analysis, and its spatial resolution was easily achievable [114]. Another example inspired by human organs (the arch of the foot) was reported by song et al., wherein a novel Janus graphene (JGF) film with concave-convex arch-shaped microstructures on both surfaces was presented [115]. The special microstructures of the graphene material could effectively hinder the full contact of two face-to-face JGF electrodes and led to a tunable pressure-dependent contact area.

**Fig. 16:**
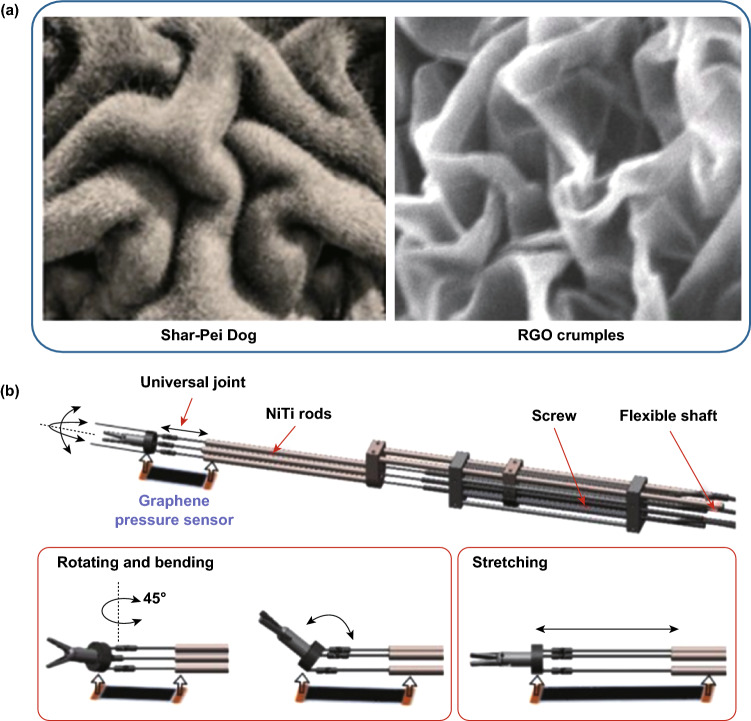
Microstructures inspired from animal and plant organs provide interesting ideas for the preparation of active materials for graphene-based tactile sensors. **a** Comparison of the surface topographies between a Shar-Pei dog’s skin and RGO crumples by using Canny edge detection. **b** Continuum surgical robots with the as-fabricated pressure sensor for the collision-aware the transoral robotic surgery procedure. Reproduced with permission from Ref. [116]. Copyright 2019 American Chemical Society

In addition to human organs, microstructures from animal and plant organs also provide interesting ideas for the preparation of active materials for graphene-based tactile sensors. A more obtrusive example is the Shar-Pei dog, and the higher dimensional patterns of Shar-Pei skin can sustain large in-plane stretching and still provide tactile perceptions so that wrinkle-crumple RGO electrodes with a high stretchability and strain-insensitive resistance profiles were fabricated by means of sequential deformation processes, as shown in Fig. 16a [116]. The stretchable pressure sensors could be integrated with two surgical robots for a transoral robotic surgery procedure. During the cadaveric testing, the RGO sensors could detect the robot-tissue contacts under joint stretches in real time to enhance the surgeon’s awareness for collision avoidance, as shown in Fig. 16b. Zhao et al. demonstrated an innovative and cost-efficient strategy to fabricate highly sensitive, stretchable, and conductive strain-sensing platforms inspired by the geometries of a spider’s slit organ and a lobster’s shell, wherein the electrically conductive composites were fabricated via embedding the 3D percolation networks of fragmentized graphene sponges (FGS) in a poly(styrene-block-butadiene-block-styrene) (SBS) matrix, followed by an iterative process of silver precursor absorption and reduction [117]. With the contribution of high stretchability from SBS and the binary synergistic effects of the designed FGS architecture and Ag NPs, a high-quality strain sensor with potential for use in E-skin applications was obtained. Inspired by an octopus’ microsuckers, Chun and coworkers developed a water-resistant and skin-adherent graphene-coated fabric (GCF) for a wearable tactile sensor, which could adhere strongly to the skin in both dry and wet environments [118]. By taking advantage of these characteristics, human physiological signals, such as wrist pulse and electrocardiography (ECG), as well as body motions and speech vibrations, could be monitored. By laminating a single-layer graphene film as the sending element on a thin polymeric support of PDMS, Chun and coworkers also achieved a peeling-resistant and water-drainable tactile sensor presenting an excellent performance under both dry and wet conditions, the construction of which was inspired by the toe pads of a tree frog [119]. Furthermore, Liu et al. [120] reported a high-performance strain sensor with a fish-scale-like graphene-sensing layer, and Jian et al. [121] presented a high-performance pressure sensor based on biomimetic aligned CNTs/graphene hierarchical structures molded from natural leaves. Inspired by mussel chemistry, Jing et al. [122] fabricated biocompatible, self-healing, highly stretchable polyacrylic acid/RGO nanocomposite hydrogel sensors by means of a dual-crosslinking mechanism including physical crosslinking and chemical crosslinking. All these examples mentioned above tell us that many wonderful hierarchal microstructures exist in nature and are waiting to be explored by researchers for fabricating tactile sensors in E-skin applications.

#### Synergy with Other Materials

With the rapid development of materials science, micronano materials with various morphologies and functions have been designed and synthesized. To advance the applications of E-skin, these materials can be integrated with versatile graphene materials in various ways to achieve effects in tactile sensors exemplifying that the combination can be greater than the sum of the individuals [33].

##### Combined with Inorganic Functional Materials

ZnO, as a common inorganic semiconductor possessing a large bandgap and exciton binding energy, an inherently high transparency and excellent luminescence at room temperature, has become a celebrated material widely used in liquid crystal displays, thin-film transistors, light-emitting diodes and other electronic products, particularly tactile sensors [48, 87]. Sun et al. [48] found that the coupling effect obtained between ZnO nanoparticles and graphene nanoplatelets could make a strain sensor exhibit perfect linearity for its whole working range. Hassan et al. [87] found that the presence of ZnO increased the connectivity between flakes of graphene, and when combined with a random micro-ridged PDMS substrate, the fabricated strain sensor achieved stretchability up to 30% and bendability down to 10 mm in diameter. Pham and coworkers constructed an exotic heterostructure pressure sensor based on ZnO/chlorine radical-trap-doped bilayer graphene, wherein the heavy p-type chlorine trap doping in the graphene channel led to chlorine radicals without damaging the graphene and made a considerable contribution to the significantly improved sensing effect [49].

**Fig. 17:**
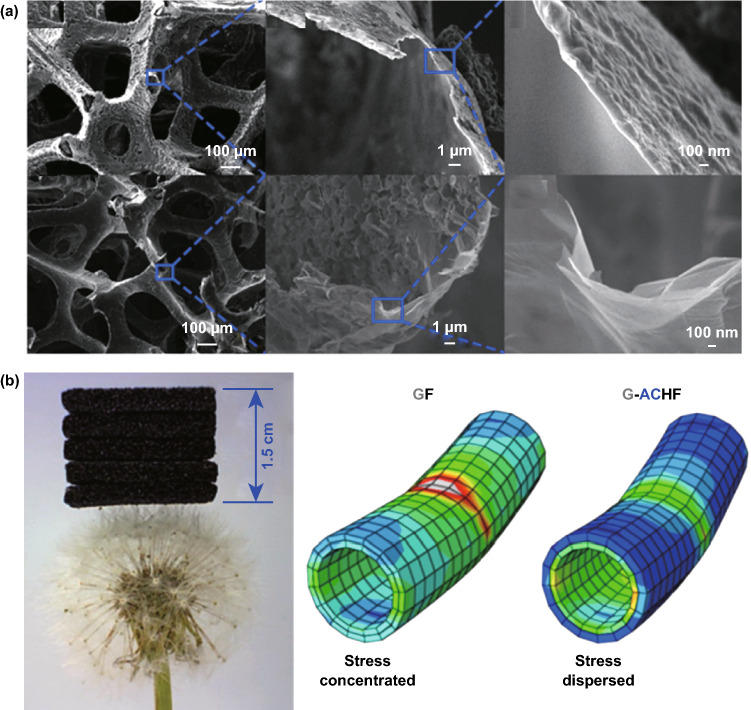
Graphene combined with inorganic functional materials to enhance the sensing performance of piezoresistive devices. **a** SEM images at different magnifications of a graphene-amorphous carbon hierarchical foam and graphene foam. **b** Five ultralight graphene-amorphous carbon hierarchical foam pieces with sizes of 20 × 20 × 3 mm^3^ stacked on the corolla of dandelion, and different simulated stress dispersion statuses of graphene foam and graphene-amorphous carbon hierarchical foam tube walls under the same line load. Reproduced with permission from Ref. [123]. Copyright 2017 American Chemical Society

In addition to ZnO, other inorganic materials can also be employed to construct functional composites to enhance the sensing performance of tactile sensors. For example, Ma et al. [123] synthesized a novel kind of ultralight graphene-amorphous carbon (AC) hierarchical foam, with an inner layer of graphene and an outer layer of AC, by CVD at 1065 °C, as shown in Fig. 17a. Owing to this unique structure, the inner graphene layer with a high conductivity and integrity provided the high sensitivity, while the outer AC layer helped to enhance the durability and mechanical resiliency, which dispersed the pressure and led to the high durability against strain, as shown in Fig. 17b. By hybridizing carbon nanofibers (CNFs) with graphene nanoplates (GNPs) within a PDMS medium, Zhang et al. [124] presented a new technique to synergistically improve a sensor’s sensitivity and cycle stability. Compared with tactile sensors containing only CNFs or GNPs, the hybridized devices exhibited a better performance with a great linear range and a substantially improved stability. By taking advantage of the different chemical potentials between graphene and Zn, current signals can be obtained spontaneously from redox-induced electricity in the presence of saline water. Inspired by this phenomenon, Wang et al. [125] fabricated a novel self-powered sensing device based on a highly stretchable graphene film and a woven meandering zinc wire. From another point of view, Shi and coworkers found that graphene hybridization could significantly strengthen CNT networks, especially at nanotube junctures, and enhance the resistance to buckling and bundling under cyclic strains up to 20% [101].

##### Combined with Polymers

As an important member of the material family, polymers present many excellent properties, such as light weight, mechanical flexibility, and their generally good solution processability, as well as a good compatibility with large-area and flexible solid supports; all of these characteristics cannot be matched by other materials and endow polymers with the ability to manufacture various sensing devices [9]. Furthermore, the inherent characteristics of polymers, such as their susceptibility to noncovalent interactions (including hydrogen bonds, charge transfer, dipole–dipole interactions, photoexcitation and reversible transformations), enable effective interactions with other materials to yield multifunctional composite materials [15].

**Fig. 18:**
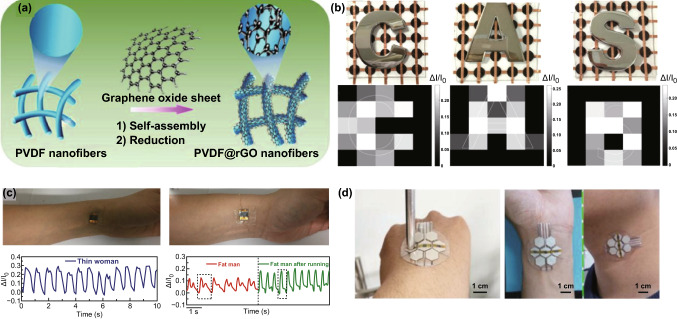
Typical paradigms concerning graphene combined with polymers to enhance the sensing performance of piezoresistive devices. **a** Schematic illustration of the mechanism for the formation of PVDF fibers coated by RGO nanosheets, followed by electrostatic interactions. **b** Top view of the metal letters “C,” “A” and “S” positioned over the pressure sensor array and the current map of pressure distributions. **c** Photograph of the device loaded on two wrists for testing blood pressure through near-surface arteries. Reproduced with permission from Ref. [128]. Copyright 2016 Elsevier Ltd. **d** The multifunctional E-skins attached on a hand, wrist and throat to monitor biosignals. Reproduced with permission from Ref. [50]. Copyright 2017 Elsevier Ltd.

Poly(vinylidene fluoridetrifluoroethylene) (P(VDF-TrFE)), as a natural elastic conductive building block, is usually functionalized with graphene materials and has widely been used in the piezoresistive sensors [50, 126, 127]. For example, Lou and coworkers first reported the fabrication of a self-assembled 3D film platform that combined a naturally viscoelastic material (P(VDF-TrFE)) with RGO by a simple, efficient two-step solution process, as shown in Fig. 18a [128]. The authors were found that the piezoresistive sensor with a sandwich structure displayed a high sensitivity, low detection limit and low working voltage, and the array could be used as highly sensitive E-skins for mapping spatial pressure distributions and monitoring human physiological signals, including real-time pulses and muscle movements, as displayed in Fig. 18b, c. Then, researchers integrated three types of sensors (a pressure sensor, photodetector and gas sensor) and three on-chip microsupercapacitors in parallel into a single pixel to construct a multifunctional self-powered E-skin system [50]. The fabricated integrated system could monitor biosignals by being worn on the human body and exhibited great mechanical flexibility while subjected different bending curvatures, as shown in Fig. 18d. Coupling the high piezoelectric coefficient of P(VDF-TrFE) with the outstanding electrical properties of graphene, the graphene/P(VDF-TrFE) heterostructure could also be used to fabricate a highly sensitive, flexible and biocompatible pressure sensor [129].

The abovementioned graphene/polymer composites were fabricated by simply mixing graphene and polymers together, hardly yielding well-defined composite materials and negatively affecting the performance of pressure-sensing devices based on such materials. To solve this problem, Lin and coworkers prepared a highly flexible self-healing conductive polymer composite consisting of graphene, poly(acrylic acid) and amorphous calcium carbonate by a biomineralization-inspired process [130]. Strain sensing based on this bioinspired dynamically crosslinked graphene/polymer composite possessed good editability and processability, and the material could be fabricated into stretchable strain sensors of various structures that worked well both in air and under water. By taking advantage of a polymer’s nature, all kinds of excellent structures with the uniform size of the 0D micro-ball can be synthesized. Based on electrostatic interactions, RGO would cover the polymer balls to produce polymer ball @RGO nanoparticles [128–131]. Due to the bending of graphene sheets by the van der Waals attractive force, the PMMA ball @ RGO-based tactile sensor, at pressures < 1 torr, showed an increased resistance value [131]. Additionally, the detecting limit of PS ball@rGO-based pressure sensors could be as low as 3 Pa with a low energy consumption of ~ 1 μW at a low bias voltage of 1 V; a fast response time of 50 ms with a high sensitivity of 50.9 kPa^−1^ at 3–1000 Pa and a high stability for 20,000 loading–unloading cycles could also be obtained [128]. Furthermore, PS balls could also be doped into RGO fragments to fabricate ultrasensitive small strain detectors [131]. The GF could be very effectively tuned by changing the size and doping ratio of the nanoparticles.

**Fig. 19:**
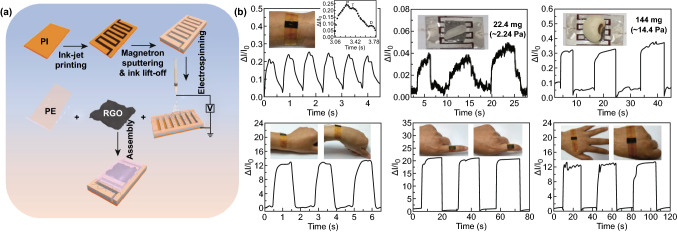
A representative graphene/polymer tactile sensor obtained by taking advantage of the amphiphilicity of GO. **a** Schematic of the fabrication of a flexible piezoresistive sensor. **b** Various practical applications, including a biomonitoring capability, loading tiny objects, and monitoring various human movement behaviors. Reproduced with permission from Ref. [135]. Copyright 2018 WILEY–VCH Verlag GmbH & Co. KGaA, Weinheim

From another point of view, due to the certain amphiphilicity caused by the hydrophilicity of the oxygen-containing groups and the hydrophobicity of *π*-conjugated graphene fragments, GO, as a novel cousin of graphene, can be considered as a 2D surfactant for use as a dispersing agent or to generate Pickering emulsions [132]. Taking into account this significance of GO in the formulation of advanced functional hybrid materials, Scaffaro et al. [133] exploited GO for poly(lactic acid)-poly(ethylene–glycol) blends. The presence of GO not only improved the mechanical properties of the composites but also endowed them a good electrical performance to obtain high-quality tactile sensors. In addition, due to GO containing abundant oxygen groups and poly(vinyl alcohol) (PVA) containing hydroxyl groups, a homogeneous dispersion of GO into PVA and strong interfacial adhesion between them could be achieved, enhancing the tensile strength, Young’s modulus and elongation at break of PVA [134]. Liu et al. [135] fabricated a flexible and highly sensitive pressure sensor based on wrinkled graphene film/innerconnected PVA nanowires/interdigital electrodes, as shown in Fig. 19a. Due to the synergistic effect between graphene and PVA, the as-prepared pressure sensor realized a high sensitivity of 28.34 kPa^−1^ and could detect subtle pulse beats and monitor various human movement behaviors in real time (Fig. 19b). In addition, different polymerization methods among polymer monomers combined with graphene can also produce excellent pressure-sensing materials [136–138]. The as-fabricated tactile sensors based on these active layers not only possessed a high pressure-sensing performance but also achieved self-healing, thermal response and other properties of human skin, attaining the ideal platform to realize the practical application of E-skin.

### Graphene Tactile Sensors Based on FET Devices

In the above, the development of pressure-sensing devices based on capacitance and piezoresistivity in recent years is introduced in detail. Nevertheless, the neighboring interference of capacitive types, the low pixel density of piezoresistive types, and the inevitable low contrast ratio and crosstalk effect of passive-matrix sensor arrays are difficult to apply to practical E-skin [139]. In recent years, field-effect transistor (FET)-type pressure sensors have attracted broad attention from a wide variety of scientific and technique communities and have become an important topic of general concern owning to their inherent properties, such as excellent signal amplification, high array uniformity, high spatial contrast and facile integration with electrical circuitry [44].

**Fig. 20:**
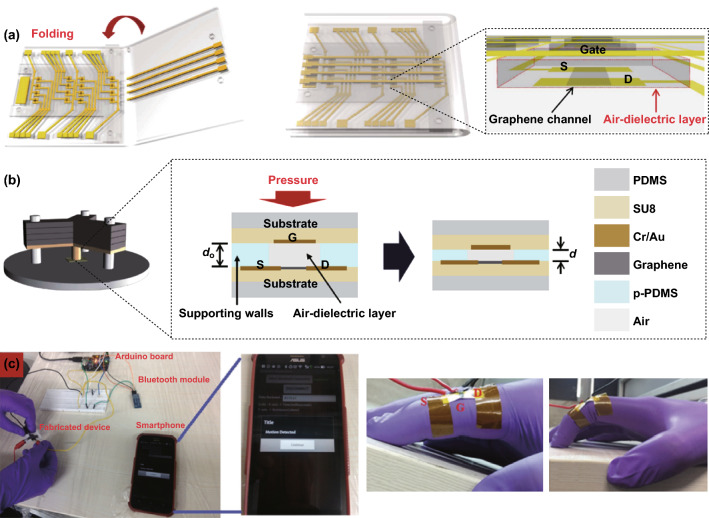
A representative graphene tactile sensors based on FET devices. **a** Schematic images of pressure-sensitive graphene FETs with air-dielectric layers before and after folding. The air-dielectric layer is placed between the graphene channel and the gate electrode, as illustrated in the schematic image (inset). **b** Schematic illustrations for the pressure-sensing mechanism using an air-dielectric graphene FET. Reproduced with permission from Ref. [45]. Copyright 2017 Macmillan Publishers Limited. **c** Integration of a graphene/MoS_2_ device with a smartphone to acquire and transfer the electronic data of human motion by Bluetooth communication. Reproduced with permission from Ref. [140]. Copyright 2018 WILEY–VCH Verlag GmbH & Co. KGaA, Weinheim

FETs generally consist of four typical parts, including the gate electrode, source and drain electrodes, dielectric layer and semiconductor active layer. The active layer, which is located in the channel between the sources and drain electrodes, is generally isolated from the gate electrode by a dielectric [45, 140]. The optimization of each part of the FET device can enhance the performance of the device and might also provide opportunities for high-quality tactile sensors. Thus far, most of the dielectric layers of FET tactile sensors have been solid species, where the charge carrier transport functionality of the semiconductor occurred mainly in a few molecular layers at the active material/dielectric interface. Not all of the solid dielectric could respond to the external force during sensing, thus, limiting the sensitivity and resolution capacity of these sensors. To address this issue, Shin et al. [45] designed an unconventional approach for fabricating fully integrated active-matrix arrays of pressure-sensitive top-gate graphene transistors with an air-dielectric layer, simply formed by folding two opposing panels (Fig. 20a). Due to the clean interface between the graphene channel and air, these air-dielectric graphene FETs displayed excellent electrical properties and a high reliability under ambient conditions. As illustrated in Fig. 20b, the height of the air gap was determined by the thickness of elastomeric partition spacers between the graphene and top gate, and it decreased by applying pressure with increasing capacitance of the metal-air-graphene structure, which could not only enhance the detection range of tactile sensors but also lead to low fabrication costs and densifications of these sensor arrays.

Another example related to reducing the efforts and cost of the fabrication techniques for the FET-based tactile sensor configuration was reported by Sahatiya and coworkers, wherein 2D graphene/MoS_2_ was used as the active layer, cellulose paper was used as the dielectric and graphite pencil trace as the gate [140]. Owing to the low-cost and biodegradability of cellulose paper, the as-fabricated graphene/MoS_2_ transistor was not only easily fabricated but also an ultrasensitive strain sensor; the graphene/MoS_2_ channel acted as a sensing layer, and the electrical resistance could be greatly varied by application of different strains. More interestingly, by interfacing the sensor with a microcontroller, the data could be acquired and transferred to a smartphone through Bluetooth communication, thus, enabling human motion monitoring, as shown in Fig. 20c. In this work, except for graphene materials, other ultrathin soft 2D materials, such as MoS_2_, are also being particularly highlighted. It should be noted that because of the downscaling limit of silicon-based devices, atomic layered 2D materials, ranging from graphene and its derivatives to transition metal dichalcogenides (TMDCs), metal carbides and nitrides (MXenes), and montmorillonite (MMT), have recently become a focus for advanced electronics [141]. From another point of view, their unique physiochemical properties along with extraordinary softness and inherent flexibility, high transparency and carrier transport properties have attracted significant interest for use in mimicking the multifunctionalities of human skin [142]. Although graphene has many physiochemical properties similar to other two-dimensional materials, there are many differences between them. Graphene possesses a unique band structure in which the valence and conduction band have an overlap at the Dirac point; thus, it simultaneously presents characteristics of a metal and a semiconductor, which allows it to be used as both electrodes and semiconductor layers in FET-based tactile sensors [143]. However, as graphene is a zero-gap semiconductor, the on/off ratio of graphene FETs has always been relatively low, limiting its applicability in tactile sensors. Because of their bandgap, TMDCs and MXenes have typical semiconductor properties and good optical properties, which provide unique characteristics hardly found in graphene [144]. However, the preparation technology of these two-dimensional materials is not as mature as that of graphene, and its commercial implementation is still a long way off. It should be noted that the contact resistance between graphene and other two-dimensional materials is substantially lower than that between a metal and two-dimensional semiconductor, greatly improving the performance of tactile sensors constructed with heterogeneous structures of graphene and other two-dimensional materials.

Although FET-based tactile sensors are generally very sensitive, easily integrated, realize real-time detection, the touch point of the abovementioned FET pressure sensors are mostly located in the gate or channel region [145]. As a result, when a large number of sensors are assembled in large-area tactile skin-type applications, high-voltage operation is needed with high power consumption, which further hinders the practical application of the devices [146, 147]. To conquer this challenge, Yogeswaran et al. [147] fabricated a low-voltage piezoelectric graphene field-effect transistor (GFET) for pressure sensors in tactile sensing, wherein a GFET was connected with a piezoelectric metal–insulator-metal (MIM) capacitor in an extended gate configuration. By taking advantage of the piezopotential generated from the piezoelectric MIM capacitor, which could modulate the channel current of the GFET, the current sensors could operate at a considerably lower voltage and exhibit a higher sensitivity. Hwang et al. [148] also fabricated a touch sensor using a piezoelectric polymer, wherein graphene was used as active layer of the FET, and the piezoelectric potential created by an externally applied force to the PVDF-TrFE layer acted as a gate modulation voltage, controlling the carrier transport across the graphene-silicon interface. The sophisticated structure not only saved energy but also improved the sensitivity of the graphene FET-based touch device by seven times.

**Fig. 21:**
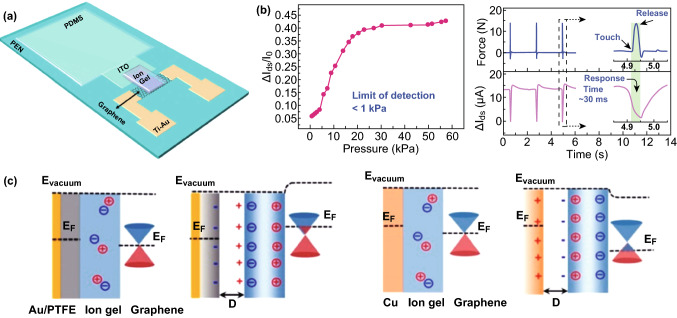
High-performance sensing devices by combining a FET with a triboelectric nanogenerator. **a** Schematic diagram of the graphene tribotronic device. **b** Characterization of the graphene tribotronic touch sensor. Reproduced with permission from Ref. [146]. Copyright 2016 WILEY–VCH Verlag GmbH & Co. KGaA, Weinheim. **c** Energy band diagram of the tribotronic GFET device in contact with poly(tetrafluoroethylene) and Cu. Reproduced with permission from Ref. [145]. Copyright 2018 American Chemical Society

In addition to piezopotential MIM capacitors, the induced triboelectric potential can also be able to couple with FETs for modulating the carrier transport in semiconductor channels and helping to obtain high-performance devices [145]. For example, a graphene tribotronic touch sensor based on the coplanar coupling of a single-electrode-mode triboelectric nanogenerator (S-TENG) and a GFET was constructed by Khan et al. [146], as shown in Fig. 21a. When any object touched the friction layer of the S-TENG, charges would be produced due to the triboelectric effect, which could act as the gate bias to modulate the channel current transport without an external gate voltage. Such as-fabricated tribotronic sensors displayed a sensitivity of ≈ 2% kPa^−1^, a limit of detection < 1 kPa, and a response time of ≈ 30 ms (Fig. 21b). Meng et al. [145] also fabricated a mechanosensation-active matrix gated by triboelectric potential, instead of applying gate voltages, and was based on a direct-contact tribotronic planar graphene transistor array, wherein an ion gel was utilized as both the dielectric layer of the FET device and the friction layer for triboelectric potential coupling to achieve highly efficient gating and sensation properties. As shown in Fig. 21c, different contact distances between the ion gel and other friction materials produced different triboelectric potentials, which were directly coupled to the graphene channel, and led to different output signals through modulating the Fermi level of graphene. As a result, the sensor array (1) exhibited excellent sensing properties, (2) could be used to recognize different categories of materials, and (3) could sense contact distances and realize a 2D color map of an object. These results suggest graphene-based FET tactile sensors have great promise in human–robot interfaces, electronic artificial skin, multifunctional sensors, and smart wearable devices.

## Summary and Outlook

Recently, targeting high-performance graphene-based tactile sensors, great progress has been achieved mainly in terms of the sophisticatedly designed morphologies of graphene and its derivatives, the working principles aiming at providing a fundamental knowledge of the sensing processes, state-of-the-art protocols targeting high-performance sensing, and the development of synergy with other materials. Indeed, the tremendous advancements accumulated to date have brought tactile sensors a significant step closer to the potential applications of flexible and wearable E-skins, such as health monitoring devices, artificial intelligence, and human–machine interfaces. Continuous efforts to further improve the overall qualities of this kind of sensor, including sensitivity, detection range, pattern recognition and spatial resolution of external forces, response time, stability and reproducibility, limit of detection, capability of digital and intelligent readouts, real-time workability, etc., remain strongly desired. To achieve this improvement, the following aspects will continue to be the major subjects of this field in our opinion.

First, the synthesis, assembly and modification of high-performance graphene materials are the basis for the fabrication of high-performance graphene-based tactile sensors. As we mentioned above, graphene and its derivative materials were usually used as conductive electrodes or sensitive materials. As electrodes, graphene has a variety of intrinsic excellent physical properties, such as a high electrical conductivity, transparency, and flexibility, which lay a good foundation for building high-performance wearable devices. However, on one hand, it is difficult to industrialize the production of large-area, high-quality materials via current production technology. On the other hand, ultrathin 2D structures are easily damaged during scratching by external forces, and the reuse of devices can only be realized through the application of complex packaging technologies. Therefore, exploring the preparation process of graphene electrodes remains the basis for commercial applications of graphene-based tactile sensors. From the point of view of sensitive materials, graphene materials are easy to assemble; are easy to modify; are easy to combine with other materials, enabling them to have diverse morphologies and properties; and can be used to improve the performance of tactile sensors. However, as a zero-bandgap semiconductor, the on/off ratio of graphene is relatively low, and the current is difficult to modulate. Although there are many ways to open the bandgap in graphene, these remain in the laboratory. Therefore, scientists should do their best to explore simpler and more effective ways to realize the commercial applications of graphene as an active material.

Second, the launch of emerging sensing mechanisms in terms of molecular engineering, supramolecular assembly, and their combination with other protocols will undoubtedly be among the most important ways to construct new types of high-performance tensile sensors. For example, a self-powered sensation matrix could be constructed by sandwiching piezoelectric polymer materials between two graphene electrodes [149, 150]. According to the principle of piezoelectric nanogenerators, the sequential multistage sensation could be substantially realized. On the other hand, a further elucidation of the underlying working principles is still a significant topic. By means of template-stripping-based nanotransfer printing, a stable nanowire array nanograting can be simply and rapidly produced to yield a plasmonic sensor [28, 151]. Such an as-fabricated device coupled to monolayer graphene exhibited an ultrahigh sensitivity to applied strain by shifts in the plasmonic-enhanced Raman spectrum. Accordingly, by taking the advantage of collaborations with other materials, optimized device structures together with a deeper understanding of the underlying working principles will not only favor the construction of next-generation qualified tactile sensors but will also afford important scientific contributions to optics, electricity and materials science, which are significant issues of general concern (Table 1).

**Table 1:** Summary of graphene material-based tactile sensors

Types of devices	Sensitivity	Response time	Limit of detection	References
*Capacitive tactile sensors*				
Micro-conformal Graphene Electrodes	7.68 kPa^−1^	30 ms	1 mg	[42]
Graphene Electrodes and Air Dielectric	6.55 kPa^−1^	70 ms	8 kPa	[52]
Suspended Graphene–Polymer Heterostructure Membranes	123 aFPa^−1^	–	80 kPa	[43]
*Piezoresistance tactile sensors (one*-*, two*- *or three*-*dimensional structures)*				
Direct Laser Scribing Polydimethylsiloxane	480 kPa^−1^	2 μs/3 μs	28 Pa	[72]
A Transparent Tactile Sensor Based on GFs/PET	0.23 mm^−1^	18.1 ms	–	[62]
(PDMS) Arrays and Reduced Graphene Oxide (rGO) Film	1.71 kPa^−1^	6 ms	0–225 Pa	[70]
Graphene/Polyethylene Terephthalate (G/PET) Film	10.80 Ω/kPa	10 ms	0–600 kPa	[63]
Graphene Oxide/PolyHIPE Foam for Pressure Sensing	2.53 kPa^−1^	15.4 ms	0.6 Pa	[106]
(GPN) Combined with Polydimethylsiloxane (PDMS)	0.09 kPa^−1^	–	0–1000 kPa	[85]
Cracked Paddy Shaped MoS_2_/Graphene Foam/Ecoflex Hybrid Nanostructures	6.06 kPa^−1^	–	0.6–7.6 kPa	[98]
RGO/Polyaniline Wrapped Sponge	0.042 kPa^−1^	96 ms	0–27 kPa	[75]
(RGOF)-Based Pressure Sensors Combination of Ultrasonic Dispersion and Freeze-Drying Methods	22.8 kPa^−1^	–	0.1 Pa	[101]
An Ultralight Sparkling Graphene Block	229.8 kPa^−1^	–	0–0.1 kPa	[102]
Graphene-Paper Pressure Sensor	0.1 kPa^−1^	60 ms	0–20 kPa	[104]
Porous Graphene Sponges	0.046 kPa^−1^	–	0.3–10 kPa	[107]
Skin-like Strain Sensors Based on Graphene/Spring-like Mesh Network	72 kPa^−1^	–	1.38 Pa	[83]
PDMS Foam Coated with Graphene Nanoplatelets	0.23 kPa^− 1^	–	10 kPa	[81]
Piezoresistive Effect of Multilayer Graphene Films on Polyester Textile	0.012 kPa^− 1^	50 ms	High as 800 kPa	[96]
*Piezoresistance tactile sensors (inspired by nature)*				
The ACNT/G and m-PDMS Films	19.8 kPa^−1^	16.7 ms	0.6 Pa	[121]
A Bioinspired Hierarchical Graphene/PDMS Array	8.5 kPa^−1^	30 ms	1 Pa	[109]
Graphene Pressure Sensor with Random Distributed Spinosum	25.1 kPa^−1^	80 ms	0–2.6 kPa	[111]
Fingerprint-Like Patterned 3D Graphene Film	110 kPa^−1^	30 ms	0.2 Pa	[113]
RGO Films with Continuous Gradient Wrinkles	178 kPa^−1^	131 ms	42 Pa	[112]
Bioinspired Microstructured Pressure Sensor Based on a Janus Graphene Film	0.736 kPa^−1^	21.5 ms	0.1 kPa	[115]
*Piezoresistance tactile sensors (synergy with other materials)*				
Transparent and Self-powered Multistagesensation Matrix	–	–	800 Pa	[149]
Large-Scale Polystyrene Ball@reduced-Graphene-Oxide Core–Shell Nanoparticles	50.9 kPa^−1^	50 ms	3–3000 Pa	[128]
P(VDF-TrFe) with an Electrically Conductive Material rGO	15.6 kPa^−1^	–	1.2 Pa	[126]
Polyvinyl Alcohol Nanowires/Wrinkled Graphene Film	28.34 kPa^−1^	–	2.24 Pa	[135]
Graphene–Polymer Nanocomposite-Based Redox-Induced Electricity	–	0.11 s	–	[125]
*Graphene tactile sensors based on FET devices*				
Integrated Arrays of Air-Dielectric Graphene Transistors	2.05 × 10 ^−4^ kPa^−1^	–	250 Pa–3 Mpa	[45]
Direct-Contact Tribotronic Planar Graphene Transistor Array	0.16 mm^−1^	15 ms	–	[145]
Solution Processed Fabrication of Graphene–MoS_2_ Transistors on Paper	–	55 ms	–	[140]
Graphene Tribotronics for Electronic Skin and Touch Screen Applications	2% kPa^−1^	30 ms	1 kPa	[146]

As we have highlighted, numerous sophisticated strategies have been proposed to achieve high-performance tactile sensors based on graphene materials. However, most of the abovementioned works address only one or a few aspects of a sensor’s quality, which is still far from mimicking human skin. Clearly, a mechanically flexible and fully integrated sensor array for multiplexed monitoring of an individual’s activities, without interrupting or limiting the user’s motions, is still a formidable challenge that cannot be realized by a single capacitive-style, piezoresistive-style or FET-style array. The combination of a couple of the state-of-the-art strategies might be among the most feasible ways to address this topic to some extent.

Finally, multifunctional tactile sensing devices capable of digital and intelligent readouts are important issues required by modern E-skin applications. Therefore, the construction of high-performance tactile sensing device arrays along with effective pattern recognition algorithms is particularly important. We believe that with the joint efforts of scientists in chemistry, physics, material science, micronano processing, computer science and other disciplines, the construction of high-performance graphene-based tactile sensing systems for potential commercial uses will soon become a reality.
